# Bioactive Alkaloids as Secondary Metabolites from Plant Endophytic *Aspergillus* Genus

**DOI:** 10.3390/molecules28237789

**Published:** 2023-11-27

**Authors:** Juntai Zhu, Lixia Song, Shengnan Shen, Wanxin Fu, Yaying Zhu, Li Liu

**Affiliations:** 1 Institute of Chinese Materia Medica, China Academy of Chinese Medical Sciences, Beijing 100700, China; zhujt@cmde.org.cn (J.Z.); lisa2008hey@163.com (L.S.); snshen@icmm.ac.cn (S.S.); fwx062@163.com (W.F.); yyzhu@icmm.ac.cn (Y.Z.); 2 Center for Medical Device Evaluation, NMPA, Beijing 100081, China; 3 State Key Laboratory for Quality Ensurance and Sustainable Use of Dao-di Herbs, Artemisinin Research Center, China Academy of Chinese Medical Sciences, Beijing 100700, China; 4 School of Life Science, Beijing University of Chinese Medicine, Beijing 100029, China

**Keywords:** alkaloids, endophytic fungi, *Aspergillus*, natural products, bioactivities

## Abstract

Alkaloids represent a large family of natural products with diverse structures and bioactivities. These compounds and their derivatives have been widely used in clinics to treat various diseases. The endophytic *Aspergillus* is a filamentous fungus renowned for its extraordinary ability to produce active natural products of high therapeutic value and economic importance. This review is the first to focus on *Aspergillus*-derived alkaloids. Through an extensive literature review and data analysis, 263 alkaloids are categorized according to their structural features into those containing cytochalasans, diketopiperazine alkaloids, quinazoline alkaloids, quinoline alkaloids, indole alkaloids, pyrrolidine alkaloids, and others. These metabolites exhibited diverse biological activities, such as antibacterial activity, cytotoxicity, anti-inflammatory activity, and α-glucosidase, ACE, and DPPH inhibitory activities. The bioactivity, structural diversity, and occurrence of these alkaloids are reviewed in detail.

## 1. Introduction

Endophytic fungi are an important class of plant-associated microorganisms that have provided a bountiful source of bioactive metabolites which benefit human health and have for decades attracted increasing attention from researchers [1,2,3]. Among them, the genus *Aspergillus* is one of the most widely studied filamentous fungi and renowned for its extraordinary productivity when it comes to active natural products with therapeutic values, making it of economic importance [4,5,6]. At present, the genus *Aspergillus* is known to comprise more than 340 species, such as the common *A. terreus*, *A. flavipes*, *A. fumigatus*, and *A. ochraceus* species [7]. These species have been reported to produce a large and chemodiverse range of metabolites, including polyketides, steroids, alkaloids, and terpenoids. These have been shown to exhibit significant anticancer, antibacterial, antifungal, and anti-inflammatory activity properties [6,8].

Alkaloids represent a large family of low-molecular-weight organic compounds containing at least one nitrogen atom. They are mainly derived from amino acids and incorporated in complex cyclic structures. To date, dozens of alkaloids have been separated from endophytic fungi and have been shown to display biodiversity [9]. Some of them have been widely applied to treat a variety of diseases [10]. Examples include vinblastine and vincristine from *Talaromyces radicus* CrP20 of *Catharanthus roseus* [11]; 9-methoxycamptothecin and 10-hydroxycamptothecin from *Fusarium solani* of *Apodytes dimidiata* E. Mey. ex Arn (Icacinaceae) [12]; camptothecin from *Entrophospora infrequens* of *Nothapodytes foetida* (well-known anticancer agents) [13], huperzine A from various endophytic fungi collected from *Huperzia* sp., and *Phlegmariurus* sp. (used as a neuroprotective agent) [14]. Thus, the alkaloids have great therapeutic and application value in clinics. It is worthy to continue to explore the alkaloids with novel structures and potent biological activities or new mechanism of action.

Alkaloids are also one of the major types of metabolites produced by *Aspergillus* species. These alkaloids possess diverse structures with significant physiological effects, including anti-inflammatory activity, antimicrobial activity, cytotoxicity, and α-glucosidase inhibition activity. According to structural features, alkaloids from *Aspergillus* are mainly divided into cytochalasans, diketopiperazine alkaloids, quinazoline alkaloids, quinoline alkaloids, indole alkaloids, and pyrrolidine alkaloids, though there are others. A number of excellent reviews on the chemical structures and biological activities of alkaloids have been published in recent years [9,10,15,16,17,18,19,20,21,22,23]. Two of these reviews are on alkaloids from *Aspergillus* genus. In 2020, Xu K., et al. summarized the chemistry and bioactivity of heterocyclic alkaloids from marine-derived *Aspergillus* species [22]. In 2021, Youssef FS et al. reviewed structures and activities of alkaloids from *Aspergillus* derived from marine organisms [23]. At present, comprehensive literature with special focus on the alkaloids derived from the plant endophytic fungi *Aspergillus* have not been retrieved. Herein, this review focuses on structural diversity and bioactivity, as well as source information of alkaloids to fill the research gap. A total of 263 alkaloids (**1**–**263**) were comprehensively summarized, including the name of the fungus from which it is derived and its host plant, as well as the compound names, chemical structures, and bioactivity of isolated metabolites. We hope that the review can provide a valuable reference for drug discovery and development of alkaloids derived from plant endophytic fungi *Aspergillus* species.

## 2. Methodology

Preparation for the present study began in May 2023, thus this review mainly presents the literature published from January 2004 to May 2023 using the PubMed and Web of Science databases. The literature search was performed using keywords endophytic fungi, *Aspergillus*, and alkaloids to retrieve information focused on the discovery of natural products. The research papers written in English, and the abstracts in English and full text in Chinese were included in this review.

## 3. Bioactive Compounds from Plant Endophytic Fungi

### 3.1. Cytochalasans

**Figure 1:**
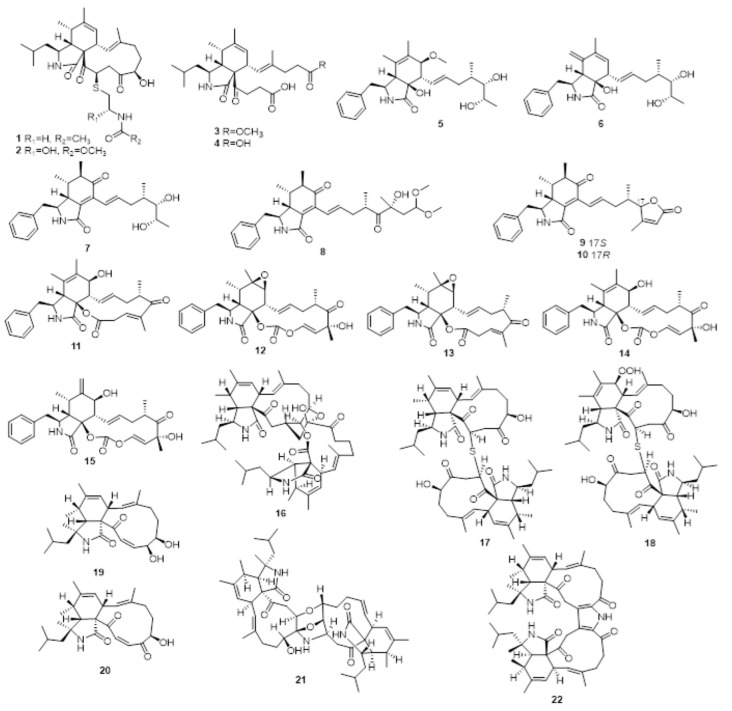
Structures of cytochalasans (**1**−**22**) produced by the endophytic fungi of the *Aspergillus* genus.

Detailed chemical research into *A. micronesiensis* from *Phyllanthus glaucus* revealed new merocytochalasans cyschalasins A (**1**) and B (**2**) (Figure 1, Table 1), as well as secochalasins A (**3**) and B (**4**). Compounds **1** and **2** possessed moderate antimicrobial activities against methicillin-resistant *Staphylococcus aureus* (MRSA), *Candida albicans*, and *S. aureus* with 50% minimum inhibitory concentration (MIC_50_) values from 10.6 ± 0.1 to 94.7 ± 1.3 μg/mL, and moderate cytotoxicities against HL60, A549, Hep3B, MCF-7 and SW480 with half maximal inhibitory concentration (IC_50_) values from 3.0 to 19.9 μM. But compounds **3** and **4** were inactive against these microbials and human cancer cell lines [24].

The endophytic fungus *Aspergillus* sp., associated with the *Pinellia ternata* tubers, produced six new seco-cytochalasins A–F (**5**–**10**), and three known cytochalasins; cytochalasin Z17 (**11**), cytochalasin E (**12**), and rosellichalasin (**13**). These isolates exhibited cytotoxicity against A549 with IC_50_ values from 7.8 to 70.2 μM. Compound **9** could reverse multidrug resistance (MDR) in a doxorubicin (DOX)-resistant human breast cancer (MCF7/DOX) cell line at 16 μM [25].

Chemical investigation of *A. terreus* IFB-E030, a fungus found on *Artemisia annua*, resulted in the identification of four known metabolites: **12**, **13**, 5,6-dehydro-7-hydroxy cytochalasin E (**14**), and Δ^6,12^-isomer of 5,6-dehydro-7-hydroxy cytochalasin E (**15**). Compounds **12**–**15** showed moderate to weak cytotoxicity against KB and HSC-T6 cells and acetylcholinesterase (AChE) [26].

The endophytic fungus *A. flavipes* KIB-536 collected from *Hevea brasiliensis* generated three homodimers, bisaspochalasins A−C (**16**−**18**), and two known isolates, aspochalasins B (**19**) and D (**20**). Compound **16** displayed human T-cell proliferation inhibitory activity with an IC_50_ of 15.8 μM, and exhibited low cytotoxic activity to T-cells [27]. In addition, *A. flavipes* KIB-392 collected from *Hevea brasiliensis* produced new bisaspochalasins D (**21**) and E (**22**). Compound **21** showed cytotoxic activity against HL-60, SMMC-7721, A-549, MCF-7, and SW-480 cells with IC_50_ values in the range of 4.45 to 22.99 μM. Compound **21** also displayed neurite-outgrowth activity for PC12 cells with a differentiation rate of 12.52% at 10 μM [28].

### 3.2. Diketopiperazine Alkaloids

**Figure 2:**
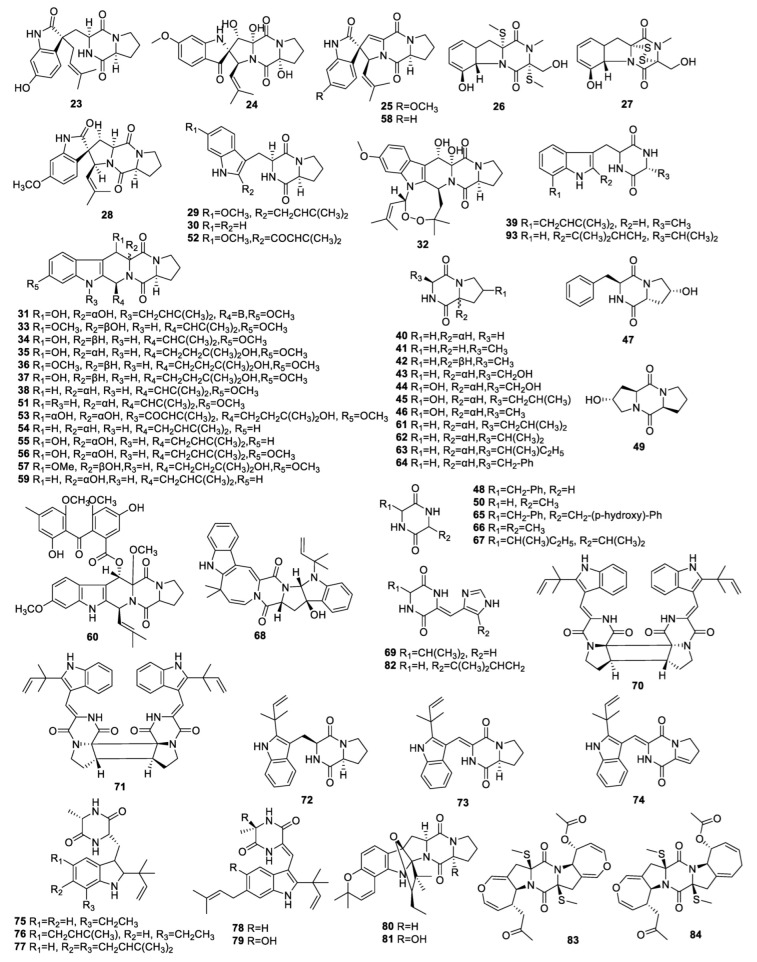
Structures of diketopiperazine alkaloids (**23**−**84**, and **93**) from endophytic fungi of the *Aspergillus* genus.

The chemical research into the endophytic fungus *A. fumigatus* from the plant stem *Erythrophloeum fordii* Oliv. (Leguminosae) revealed a new compound, spirotryprostatin K (**23**) (Figure 2, Table 2), and two known compounds, spiro[5H,10H-dipyrrolo[1,2-a:1′,2′-d]pyrazine-2(3H),2′-[2H]-indole]-3′,5,10(1′H) trione (**24**) and 6-methoxyspirotryprostatin B (**25**). None of them inhibited nitric oxide (NO) production with IC_50_ values beyond 10 μM [29]. Chemical investigation into *A*. *fumigatus* D, an endophyte which grows on *Edgeworthia chrysantha* Lindl., resulted in the isolation of **25**, bisdethiobis(methylthio)gliotoxin (**26**), gliotoxin (**27**), and spirotryprostatin A (**28**). Compounds **25** and **26** displayed potent inhibitory activity against *C. albicans* with the same MIC of 0.39 μg/mL. Compound **28** demonstrated the strongest inhibition on *S. aureus* and *Escherichia coli* with the same MIC of 0.39 μg/mL [30].

The endophytic fungus *A. fumigatus* LN-4 separated from the stem bark of *Melia azedarach* generated 24 natural products containing **24**, **26**, tryprostatin A (**29**), brevianamide F (**30**), fumitremorgin B (**31**), verruculogen (**32**), cyclotryprostatin B (**33**), cyclotryprostatin A (**34**), verruculogen TR-2 (**35**), 12β-hydroxy-13α-methoxyverruculogen TR-2 (**36**), and 12β-hydroxyverruculogen TR-2 (**37**), fumitremorgin C (**38**), terezine D (**39**), and cyclo-(Pro-Gly) (**40**), cyclo-(Pro-Ala) (**41**), cyclo(D-Pro-L-Ala) (**42**), cyclo-(Pro-Ser) (**43**), cyclo-(Ser-trans-4-OH-Pro) (**44**), cyclo-(Leu-4-OH-Pro) (**45**), cyclo-(Alatrans-4-OH-Pro) (**46**), cyclo-(cis−OH-D-Pro-L-Phe) (**47**), cyclo-(Gly-Phe) (**48**), cyclo-(Pro-trans-4-OH-Pro) (**49**), and cyclo-(Gly-Ala) (**50**) [31]. Continuing research on the fungus *A. fumigatus* LN-4 using the one strain many compounds (OSMAC) method, compound **25**, 12α-fumitremorgin C (**51**), and 18-oxotryprostatin A (**52**) were also identified [32]. Compounds **24**, **31**, **32**, and **38** exhibited antifeedant activity against armyworm larvae with antifeedant indexes (AFI) of 5.0%, 50%, 55.0%, and 15.0%, respectively. Compounds **26**, **29**−**33**, **35**−**38**, and **46** showed significant and weak toxicities against brine shrimps with median lethal concentration (LC_50_) values of 13.6−83.7 μg/mL. Compound **30** exhibited inhibition on turnip (*Raphanus sativus*) shoots and root elongation with a response index (RI) of −0.76 and −0.70 at 120 ppm, respectively, and possesses a potent inhibitory effect on amaranth (*Amaranthus mangostanus*) seedling growth with high RI of –0.9 at 40 ppm. Compounds **31**, **32**, and **36** displayed antifungal activity, with MIC values from 6.25 to 50 μg/mL [31,32].

A new metabolite, asperfumigatin (**53**), together with nine known compounds, **30**, **35**, **38**, demethoxyfumitremorgin C (**54**), cyclotryprostatin C (**55**), 12,13-dihydroxyfumitremorgin C (**56**), 20-hydroxycyclotryprostatin B (**57**), spirotryprostatin B (**58**), and 13-dehydroxycyclotryprostatin C (**59**) were separated from *A. fumigatus*, an endophyte associated with the Chinese liverwort, *Heteroscyphus tener* (Steph.) Schiffn. All isolates displayed weak to moderate cytotoxicity against PC3, PC3D, A549, and NCI-H460 cells [33].

A chemical study of *A. fumigatus* associated with *Diphylleia sinensis* L. generated a new compound, fumitremorgin D (**60**), which exhibited thin cytotoxicity on HepG2 with an IC_50_ value of 47.5 μM [34].

Seven alkaloids—3-isobutypyrrolopiperazine-2,5-dione (**61**), 3-isopropyl-pyrrolopiperazine-2,5-dione (**62**), 3-seco-butyl-pyrrolopiperazine-2,5-dione (**63**), 3-benzyl-pyrrolopiperrazine-2,5-dione (**64**), 3-benzyl-6-(p-hydroxy benzyl) piperazine-2,5-dione (**65**), 3,6-dimethylpiperazine-2,5-dione (**66**), and 3-isobutyl-6-isopropylpiperazine-2,5-dione (**67**)—were separated from an endophytic *Aspergillus* sp. TPXq isolated from *Saussurea medusa*.

All compounds showed weak cytotoxicity against A549 and MCF-7 cell lines with IC_50_ values beyond 50 μg/mL [35].

The known compounds okaramine A (**68**) and JBIR 75 (**69**) were isolated from the endophyte *A. aculeatus* associated with leaves of the papaya plant *Carica papaya*. None of them showed cytotoxicity against the L5178Y mouse lymphoma cell line at 10 μg/mL [36].

The endophytic fungus *Aspergillus* sp. SK-28 isolated from the leaves of a mangrove plant, *Kandelia candel*, was fermented and yielded (−)-and (+)-asperginulin A (**70** and **71**), along with three known alkaloids, deoxybrevianamide E (**72**), brevianamides V (**73**), and K (**74**). Compound **71** and **72** showed antifouling activity against the barnacle *Balanus reticulatus* [37].

The known compounds echinulin (**75**), tardioxopiperazine B (**76**), arestrictin A (**77**), neochinulin D (**78**), and variecolorin O (**79**) were identified in *A. amstelodami* derived from marine white beans. Compounds **75**, **76, 78**, and **79** inhibited melanin production in B16 cells with IC_50_ values of 98.0 ± 1.16, 30.8 ± 5.57, 112.0 ± 0.22, and 38.5 ± 6.08 µM, respectively. None of them led to any allergic activity in RBL-2H3 cells [38].

Research into endophyte *Aspergillus* sp. GZWMJZ-258 derived from *Garcinia multiflora* (Guttiferae) led to three new indolyl diketopiperazines, gartryprostatins A−C (**80**−**82**), which displayed inhibitory activity against MV4-11 cells with IC_50_ values of 7.2, 10.0, and 0.22 μM, respectively [39].

Research on the endophytic fungus *Aspergillus* sp. (w-6) which grows on *Acanthus ilicifolius* resulted in the isolation of two compounds that have been previously reported, acetylaranotin (**83**) and acetylapoaranotin (**84**) [40]. Scetylapoaranotin (**84**) was isolated from the endophytic fungus, *A. terreus* IFB-E030 collected from *Artemisia annua*, and exhibited slight inhibitory activity against KB cells, HSC-T6 cells and AChE, with IC_50_ values of 71.4 ± 15.6, 144.2 ± 11.9 and 127.4 ± 17.3 μM, respectively [26].

**Figure 3:**
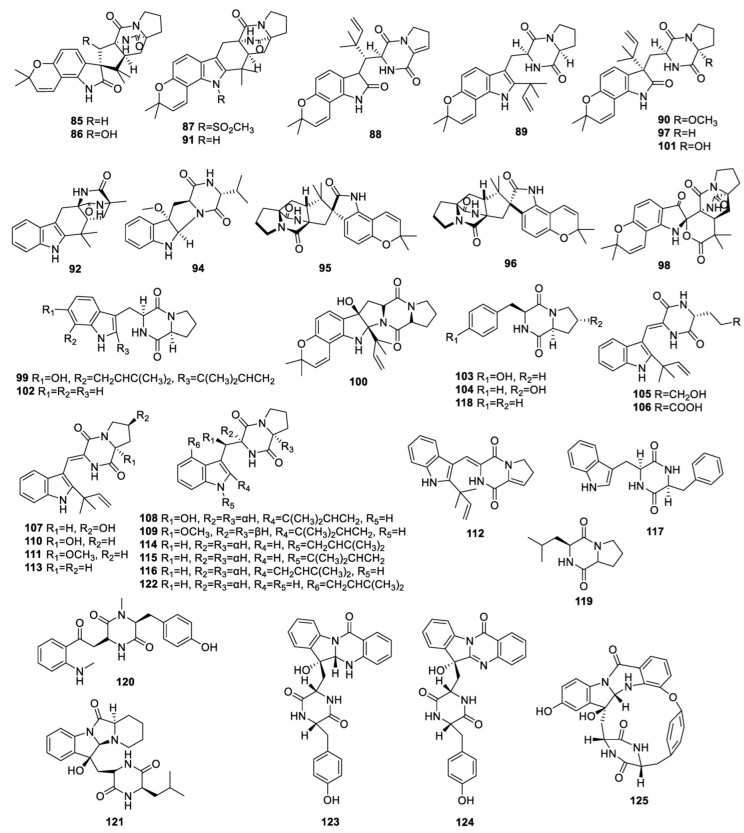
Structures of diketopiperazine alkaloids (**85**−**92**, and **94**−**125**) from endophytic fungi of the *Aspergillus* genus.

The known compounds notoamide B (**85**) (Figure 3) and selerotiamide (**86**), isolated from the endophyte *A. ochraceus*, which grows on the marine brown alga *Sargassum kjellmanianum*, did not demonstrate any antimicrobial activity against *S. aureus*, *E. coli*, or *A. niger* [41].

The detailed chemical investigation for endophyte *A. versicolor* F210, associated with the bulbs of *Lycoris radiate*, generated a new alkaloid, 21-epi-taichunamide D (**87**), along with four known analogues: dehydronotoamide C (**88**), notoamide E (**89**), notoamide Q (**90**), and (+)-stephacidine A (**91**). Compound **87** showed cytotoxicity against HL60 and A549 cells with IC_50_ values of 26.8 and 36.5 μM, respectively. Compound **90** displayed cytotoxicity against HL60 and SW480 with IC_50_ values of 19.2 and 25.5 μM, respectively. Other compounds were inactive against HL60, SMMC7721, A549, MCF7, SW480, and NCM460 cells with IC_50_ values beyond 40 μM [42].

The endophyte *A. cristatus* collected from *Pinellia ternate* tubers was studied and revealed three new alkaloids, aspergillines A–C (**92**–**94**). None of them inhibited *Bacillus subtilis* and *S. aureus* [43].

The known compounds **85**, versicolamide B (**95**), taichunamide E (**96**), and notoamide C (**97**) were separated from the moss endophyte *Aspergillus* sp. Compound **85** exhibited obvious inhibition of lipopolysaccharide (LPS)-induced NO production in RAW 264.7; the IC_50_ value was 49.85 μM [44].

An investigation into endophyte *Aspergillus* sp. Y-2 harbored on needles of *Abies beshanzuensis* led to the identification of a new compound, beshanzuamide A (**98**), together with five known isolates: **72**, **85**, **89**, **91**, and asperochramide A (**99**). None of the metabolites displayed any obvious activity against A549 or HeLa cells with IC_50_ values beyond 50 μM [45].

Six known alkaloids—**72**, **95**, **97**, notoamide D (**100**), notoamide M (**101**), and cyclo (D-Pro-L-Trp) (**102**)—were acquired from the *Nicotiana tabacum*-derived fungus *A. versicolor*. All compounds exhibited anti-mosaic virus (TMV) activity with IC_50_ values from 22.8 to 45.6 μM [46].

A study on *Aspergillus* sp. 87 derived from mangrove led to the isolation of compounds **28**, **30**, **58**, cyclo(L-Pro-L-tyr) (**103**), and cyclo-trans-4-OH-(L)-Pro-(L)-Phe (**104**). None of them displayed antibacterial activity against *E. coli*, *S. aureus*, *Acinetobacter baumannii*, or *Pseudomonas aeruginosa* [47]. Five new alkaloids, aspergiamides A–E (**105**–**109**), and eight known compounds—**30**, brevianamide Q (**110**), brevianamide R (**111**), brevianamide K (**112**), brevianamide W (**113**), N-prenyl-cyclo-L-tryptophyl-L-proline (**114**), epi-deoxybrevianamide E (**115**), and cyclo-(tryptophyl-phenylalanyl) (**116**)—were identified from the mangrove endophyte *Aspergillus* sp. 16-5c. Compounds **105**, **107**, and **112**–**114** displayed *α*-glucosidase inhibition, with IC_50_ values from 7.6 to 83.9 µM. None of the compounds exhibited significant inhibition of protein tyrosine phosphatase 1B (PTP1B) enzyme [48].

The sea cucumber-derived fungus *A. fumigatus* M580 was cultivated and the known compounds **25**, **26**, **30**, **56**, tryprostatin B (**117**), cyclo(L-prolinyl-L-phenylalanine) (**118**), and cyclo(Lprolinyl-L-valine) (**119**) were obtained. Compound **117** clearly inhibited *Enterococcus faecalis*, with an MIC value of 64 µg/mL. Compound **118** indicated α-glucosidase inhibition with an inhibition rate of 10.3 ± 0.8% at 100 μg/mL [49].

The endophyte *Aspergillus* sp. HAB10R12, obtained from the roots of *Garcinia scortechinii*, was fermented on potato dextrose agar (PDA), yielding three new alkaloids—aspergillinine A (**120**), C (**121**) and D (**122**)—none of which demonstrated cytotoxicity against HepG2 and A549 cells [50].

Comprehensive chemical research into *Aspergillus* sp., derived from the stem bark of *Melia azedarach* L revealed three new compounds, aspertryptanthrins A–C (**123**–**125**), which exhibited no cytotoxicity against U-2OS, MCF-7, HepG2 or HeLa cells at 50 μM [51].

**Table 2:** Diketopiperazine Alkaloids from endophytic fungi of *Aspergillus* genus and their biological activities, metabolite class, fungus, host plant(s), reference.

Fungus	Host Plant(s)	Compounds Isolated	Biological Target	Biological Activity	Reference
*A. fumigatus*	*Erythrophloeum fordii* Oliv. (Leguminosae)	Spirotryprostatin K (**23**)	Inhibitory activity on NO production	IC_50_ > 10 μM	[29]
	*Erythrophloeum fordii* Oliv. (Leguminosae)/*Melia azedarach* L.	Spiro[5H,10H-dipyrrolo[1,2-a:1′,2′-d]pyrazine-2(3H),2′-[2H]-indole]-3′,5,10(1′H) trione (**24**)	Antifeedant activity against armyworm larvae	AFI of 5.0%	[29,31],
*A. fumigatus/A. fumigatus D/A. fumigatus LN-4/A.fumigatus* M580	*Erythrophloeum fordii* Oliv. (Leguminosae)/*Edgeworthia chrysantha Lindl./Melia azedarach* L./sea cucumber	6-Methoxyspirotryprostatin B (**25**)	Inhibitory activity against *E. coli*, *S. aureus,* and *C. albicans*	MIC, 12.5, >25, 0.39 μg/mL	[29,30,31,49],
*A. fumigatus D/A. fumigatus* M580	*Edgeworthia chrysantha Lindl./*sea cucumber	Bisdethiobis(methylthio)gliotoxin (**26**)	Inhibitory activity against *E. Coli, S. aureus, C. albicans*; Toxicities against Brine Shrimps	MIC, >25, 0.78, 0.39 μg/mL; LC_50_ of 50%;	[30,49]
*A. fumigatus D*	*Edgeworthia chrysantha Lindl.*	Gliotoxin (**27**)	Inhibitory activity against *E. Coli, S. aureus, C. albicans*;	MIC, 0.78, 6.25, >25 μg/mL	[30]
*A. fumigatus D/A. fumigatus LN-4/Aspergillus* sp. 87	*Edgeworthia chrysantha Lindl./Melia azedarach L/*mangrove	Spirotryprostatin A (**28**)		MIC, 0.39, 0.39, 0.78 μg/mL	[30,31,47]
*A. fumigatus LN-4*	*Melia azedarach L*	Tryprostatin A (**29**)	Allelopathic activity against lettuce (*Lactuca sativa*) with response index (RI) of germination rates, root and shoot elongation at 200 ppm; Toxicities against brine shrimps with median lethal concentration (LC_50_);	RI of 0.82 ± 0.06, −0.13 ± 0.00 and −0.17 ± 0.13, respectively; LC_50_ of 44.8 μg/mL	[31,47,48,49]
*A. fumigatus LN-4/Aspergillus* sp. 87/*Aspergillus* sp. 16-5c/*A. fumigatus* M580	*Melia azedarach L/*mangrove/Mangrove/Sea cucumber	Brevianamide F (**30**)		RI of 0.54 ± 0.08, −0.91 ± 0.01, and −0.88 ± 0.02, respectively LC_50_ of 83.7 μg/mL	
*A. fumigatus LN-4*	*Melia azedarach* L.	Fumitremorgin B (**31**)	Allelopathic activity against lettuce (*Lactuca sativa*) with response index (RI) of germination rates, root and shoot elongation at 200 ppm; Toxicities against brine shrimps with median lethal concentration (LC_50_);	RI of 0.63 ± 0.06, −0.32 ± 0.02, −0.36 ± 0.07, respectively; LC_50_ of 13.6 μg/mL	[31]
		Verruculogen (**32**)		RI of 0.79 ± 0.08, 0.08 ± 0.03, 0.41 ± 0.01, respectively; LC_50_ of 15.8 μg/mL	
		Cyclotryprostatin B (**33**)		RI of 0.74 ± 0.06, −0.33 ± 0.02, 0.00 ± 0.00, respectively; LC_50_ of 37.9 μg/mL	
		Cyclotryprostatin A (**34**)		RI of 0.74 ± 0.06, 0.03 ± 0.02, and −0.21 ± 0.07, respectively; LC_50_ > 100 μg/mL	
		Verruculogen TR-2 (**35**)		RI of 0.85 ± 0.06, −0.25 ± 0.01, 0.21 ± 0.02, respectively; LC_50_ of 26.9 μg/mL	
		12β-Hydroxy-13α-methoxyverruculogen TR-2 (**36**)		RI of 0.85 ± 0.06, 0.04 ± 0.01, 0.19 ± 0.03, respectively; LC_50_ of 60.7 μg/mL	
		12β-Hydroxyverruculogen TR-2 (**37**)		RI of 0.78 ± 0.00, −0.21 ± 0.01, −0.05 ± 0.01, respectively; LC_50_ of 73.2 μg/mL	
		Fumitremorgin C (**38**)		LC_50_ of 40.5 μg/mL	
		Terezine D (**39**)		LC_50_ > 100 μg/mL	
		Cyclo-(Pro-Gly) (**40**)		LC_50_ > 100 μg/mL	
		Cyclo-(Pro-Ala) (**41**)		LC_50_ > 100 μg/mL	
		Cyclo(D-Pro-L-Ala) (**42**)		LC_50_ > 100 μg/mL	
		Cyclo-(Pro-Ser) (**43**)		LC_50_ > 100 μg/mL	
		Cyclo-(Ser-trans-4-OH-Pro) (**44**)		LC_50_ > 100 μg/mL	
		Cyclo-(Leu-4-OH-Pro) (**45**)		LC_50_ > 100 μg/mL	
		Cyclo-(Ala-trans-4-OH-Pro) (**46**)		LC_50_ of 66.1 μg/mL	
		Cyclo-(Cis−OH-D-Pro-L-Phe) (**47**)		LC_50_ > 100 μg/mL	
		Cyclo-(Gly-Phe) (**48**)		LC_50_ > 100 μg/mL	
		Cyclo-(Pro-trans-4-OH-Pro) (**49**)		LC_50_ > 100 μg/mL	
		Cyclo-(Gly-Ala) (**50**)		LC_50_ > 100 μg/mL	
		12α-Fumitremorgin C (**51**)		RI: 0.63 ± 0.06, 0.03 ±0.01, 0.20 ± 0.02, respectively	
		18-Oxotryprostatin A (**52**)		RI: 0.82 ± 0.06, −0.06 ± 0.02, −0.34 ± 0.09, respectively	
*A. fumigatus*	*Heteroscyphus tener* (Steph.)Schiffn	Asperfumigatin (**53**)	Cytotoxicity against PC3, PC3D, A549, and NCI-H460	IC_50_, 30.6 ± 0.2, >40, >40, >40 μM	[33]
		Demethoxyfumitremorgin C (**54**)		IC_50_, 32.0 ± 0.5, >40, >40, >40 μM	
		Cyclotryprostatin C (**55**)		IC_50_, 33.9 ± 0.2, >40, >40, >40 μM	
*A. fumigatus/A. fumigatus* M580	*Heteroscyphus tener* (Steph.)Schiffn/sea cucumber	12,13-Dihydroxyfumitremorgin C (**56**)		IC_50_, 36.2 ± 0.4, 39.6 ± 1.0, >40, >40 μM	[33,49]
*A. fumigatus*	*Heteroscyphus tener* (Steph.)Schiffn	20-Hydroxycyclotryprostatin B (**57**)		IC_50_, 32.5 ± 0.8, >40, >40, >40 μM	[33]
*A. fumigatus/Aspergillus* sp. 87	*Heteroscyphus tener* (Steph.)Schiffn/mangrove	Spirotryprostatin B (**58**)		IC_50_, 35.2 ± 0.5, >40, >40, >40 μM	[33,47]
*A. fumigatus*	*Heteroscyphus tener* (Steph.)Schiffn	3-Dehydroxycyclotryprostatin C (**59**)		IC_50_, 35.9 ± 0.6, 39.9 ± 1.3, >40, >40 μM	[34]
*A. fumigatus*	*Diphylleia sinensis*	Fumitremorgin D (**60**)	Cytotoxicity on HepG2	IC_50_, 47.5 μM	
*Aspergillus* sp. TPXq	*Saussurea medusa*	3-Isobutypyrrolopiperazine-2,5-dione (**61**)	Cytotoxicities against A549 and MCF-7 cell lines	IC_50_ > 50 μg/mL	[35]
		3-Isopropyl-pyrrolopiperazine-2,5-dione (**62**)			
		3-Seco-butyl-pyrrolopiperazine-2,5-dione (**63**)			
		3-Benzyl-pyrrolopiperrazine-2,5-dione (**64**)			
		3-Benzyl-6-(p-hydroxy benzyl) piperazine-2,5-dione (**65**)			
		3,6-Dimethylpiperazine-2,5-dione (**66**)			
		3-Isobutyl-6-isopropylpiperazine-2,5-dione (**67**)			
*A. aculeatus*	*Carica papaya*	Okaramine A (**68**)	Cytotoxity against L5178Y mouse lymphoma cell line	IC_50_ > 50 μg/mL	[36]
		JBIR 75 (**69**)			
*Aspergillus* sp. SK-28	*Kandelia candel*	(−)-Asperginulin A (**70**)	Antifouling activity against the barnacle *Balanus reticulatus*	Inactive	[37]
		(+)-Asperginulin A (**71**)		Antifouling activity	
*Aspergillus* sp. SK-28/*Aspergillus* sp. Y-2/*A. versicolor*	*Kandelia candel/Abies beshanzuensis/Nicotiana tabacum*	Deoxybrevianamide E (**72**)	Antifouling activity against the barnacle *Balanus reticulatus*; Anti-TMV activities	Antifouling activity; IC_50_ of 38.7 µM	[37,45,46]
*Aspergillus* sp. SK-28	*Kandelia candel*	Brevianamide V (**73**)	Antifouling activity against the barnacle *Balanus reticulatus*	Inactive	[37]
		Brevianamide K (**74**)			
*A. amstelodami*	Marine white beans	Echinulin (**75**)	Inhibition of melanin production in B16 cells	IC_50_ of 98.0 ± 1.16 µM	[38]
		Tardioxopiperazine B (**76**)		IC_50_ of 30.8 ± 5.57 µM	
		Arestrictin A (**77**)		-	
		Neochinulin D (**78**)		IC_50_ of 112.0 ± 0.22 µM	
		Variecolorin O (**79**)		IC_50_ of 38.5 ± 6.08 µM	
*Aspergillus* sp. GZWMJZ-258	*Garcinia multiflora* (Guttiferae)	Gartryprostatin A (**80**)	Inhibitory activity against MV4-11 cells	IC_50_ of 7.2 μM	[39]
		Gartryprostatin B (**81**)		IC_50_ of 10.0 μM	
		Gartryprostatin C (**82**)		IC_50_ of 0.22 μM	
*Aspergillus* sp. (w-6)	*Acanthus ilicifolius*	Acetylaranotin (**83**)	-	-	[40]
*Aspergillus* sp. (w-6)/*A. terreus* IFB-E030	*Acanthus ilicifolius/Artemisia annua*	Acetylapoaranotin (**84**)	Cytotoxic activity against KB and HSC-T6 cell lines; AChE inhibition	IC_50_ of 71.4 ± 15.6, 144.2 ± 11.9 μM IC_50_ of 127.4 ± 17.3 μM	[26,40]
*A. ochraceus/Aspergillus* sp/*Aspergillus* sp. Y-2	*Sargassum kjellmanianum/*moss/*Abies beshanzuensis*	Notoamide B (**85**)	Inhibition on LPS-induced NO production in RAW 264.7; Antimicrobial activity of *Staphylococcus aureus*, *Escherichia coli*, and *A. niger*	IC_50_ of 49.85 μM; Inactive	[41,44,45]
*A. ochraceus*	*Sargassum kjellmanianum*	Selerotiamide (**86**)	antimicrobial activity of *Staphylococcus aureus*, *Escherichia coli*, and *A. niger*	Inactive	[41]
*A. versicolor* F210	*Lycoris radiate*	21-Epi-taichunamide D (**87**)	Cytotoxicity against HL60 and A549	IC_50_ of 26.8 and 36.5 μM	[42]
		Dehydronotoamide C (**88**)	Cytotoxicity against HL60, SMMC7721, A549, MCF7, SW480, and NCM460	IC_50_ > 40 μM	[42]
*A. versicolor* F210/*Aspergillus* sp. Y-2	*Lycoris radiate/Abies beshanzuensis*	Notoamide E (**89**)			[42,45]
*A. versicolor* F210	*Lycoris radiate*	Notoamide Q (**90**)	Cytotoxicity against HL60 and SW480 with	IC_50_ of 19.2 and 25.5 μM, respectively	[42]
*A. versicolor* F210/*Aspergillus* sp. Y-2	*Lycoris radiate/Abies beshanzuensis*	(+)-Stephacidine A (**91**)	Cytotoxicity against A549 and the human cervical carcinoma HeLa cells	IC_50_ > 50 μM	[42,45]
*A. cristatus*	*Pinellia ternate*	Aspergilline A (**92**)	Inhibition against *Bacillus subtilis* and *Staphylococcus aureus*	Inactive	[43]
		Aspergilline B (**93**)			
		Aspergilline C (**94**)			
*Aspergillus* sp.	Moss	Versicolamide B (**95**)	Inhibition on LPS-induced NO production in RAW 264.7; Anti-TMV activities	Inactive; IC_50_ of 40.2 μM	[44,46]
		Taichunamide E (**96**)	Inhibition on LPS-induced NO production in RAW 264.7	Inactive;	[44]
		Notoamide C (**97**)	Inhibition on LPS-induced NO production in RAW 264.7; Anti-TMV activities	Inactive; IC_50_ of 36.4μM	[44,46]
*Aspergillus* sp. Y-2	*Abies beshanzuensis*	Beshanzuamide A (**98**)	Cytotoxicity against A549 and the human cervical carcinoma HeLa cells	IC_50_ > 50 μM	[45]
		Asperochramide A (**99**)			
*A. versicolor*	*Nicotiana tabacum*	Notoamide D (**100**)	Anti-TMV activities	IC_50_ of 33.6 μM	[46]
		Notoamide M (**101**)		IC_50_ of 22.8 μM	
		Cyclo (D-Pro-L-Trp) (**102**)		IC_50_ of 45.6 μM	
*Aspergillus* sp. 87	Mangrove	Cyclo(L- Pro- L- tyr) (**103**)	Antibacterial activities against *Escherichia coli*, *Staphylococcus aureus*, *Acinetobacter baumannii*, and *Pseudomonas aeruginosa*	Inactive	[47]
		Cyclo-trans-4-OH-(L)-Pro-(L)-Phe (**104**)			
*Aspergillus* sp. 16-5c	Mangrove	Aspergiamide A (**105**)	Inhibitory activities against α-glucosidase (IC_50_); PTP1B Inhibition Ratio (%)	IC_50_ of 18.2 μM; Inhibition Ratio of 20% at 100 μg/mL	[48]
		Aspergiamide B (**106**)		IC_50_ of 130.7 μM; Inhibition Ratio, <10% at 100 μg/mL	
		Aspergiamide C (**107**)		IC_50_ of 83.9 μM; Inhibition Ratio, <10% at 100 μg/mL	
		Aspergiamide D (**108**)		IC_50_ of 144.2 μM; Inhibition Ratio, <10% at 100 μg/mL	
		Aspergiamide E (**109**)		IC_50_ of 1093.5 μM; Inhibition Ratio, <10% at 100 μg/mL	
		Brevianamide Q (**110**)		IC_50_ of 198.2 μM; Inhibition Ratio, <10% at 100 μg/mL	
		Brevianamide R (**111**)		IC_50_ of 364.3 μM; Inhibition Ratio, <10% at 100 μg/mL	
		Brevianamide K (**112**)		IC_50_ of 7.6 μM; Inhibition Ratio, <10% at 100 μg/mL	
		Brevianamide W (**113**)		IC_50_ of 40.7 μM; Inhibition Ratio, <10% at 100 μg/mL	
		N-Prenyl-cyclo-L-tryptophyl-L-proline (**114**)		IC_50_ of 353.2 μM; Inhibition Ratio, <10% at 100 μg/mL	
		Epi-deoxybrevianamide E (**115**)		IC_50_ of 480.5 μM; Inhibition Ratio, <10% at 100 μg/mL	
		Cyclo-(tryptophyl-phenylalanyl) (**116**)		IC_50_ of 353.2 μM; Inhibition Ratio, <10% at 100 μg/mL	
*A. fumigatus* M580	Sea cucumber	Tryprostatin B (**117**)	Inhibition on *Enterococcus faecalis*	MIC of 64 µg/Ml;	[49]
		Cyclo(L-prolinyl-L-phenylalanine) (**118**)	α-Glucosidase inhibition	Inhibiting rate of 10.3 ± 0.8% at 100 μg/Ml;	
		Cyclo(Lprolinyl-L-valine) (**119**)	Antimicrobial activity	Inactive	
*Aspergillus* sp. HAB10R12	*Garcinia scortechinii*	Aspergillinine A (**120**)	Cytotoxicity against HepG2 and A549 cells	IC_50_ > 30 μM	[50]
		Aspergillinine C (**121**)			
		Aspergillinine D (**122**)			
*Aspergillus* sp.	*Melia azedarach* L.	Aspertryptanthrin A (**123**)	Cytotoxicity against U-2OS, MCF-7, HepG2 and HeLa cells	IC_50_ > 50 μM	[51]
		Aspertryptanthrin C (**124**)			
		Aspertryptanthrin D (**125**)			

“-” not test.

### 3.3. Quinazoline Alkaloids

**Figure 4:**
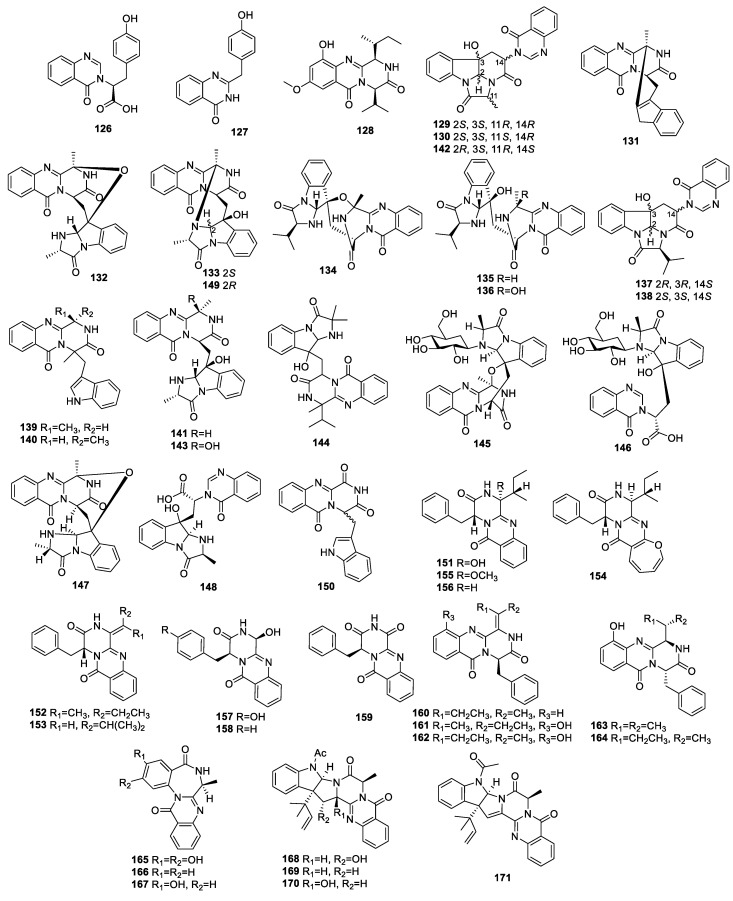
Structures of diketopiperazine alkaloids (**126**−**171**) from endophytic fungi of the *Aspergillus* genus.

Two alkaloids, asperflaloid A (**126**) (Figure 4, Table 3) and 2-(4-hydroxybenzyl)quinazolin-4(3H)one (**127**), were obtained from *A. flavipes* DZ-3, derived from twigs of *Eucommia ulmoides* Oliver. Compound **127** showed α-glucosidase inhibition with an IC_50_ value of 750.8 µM [52].

A new quinazoline derivative, versicomide E (**128**), was identified from the moss endophytic fungus *Aspergillus* sp. This compound was not found to exhibit anti-inflammatory activity to suppress NO production induced by LPS in RAW 264.7 cells [44].

The known alkaloid isochaetominine (**129**), from the mangrove-derived fungus *A.* sp. 87, was devoid of antibacterial activity against *P. aeruginosa*, *S. aureus*, *A. baumannii*, and

*E. coli*, with MIC values beyond 100 µM [47]. Chaetominine (**130**) was separated from *Saussurea medusa*-derived endophyte *Aspergillus* sp. TPXq. The IC_50_ values of **130** against A549 and MCF-7 tumor cells were 0.18 and 0.89 μg/mL, respectively [35].

As well as the metabolites **129** and **130**, fumiquinazoline J (**131**) and fumiquinazoline C (**132**) were also isolated from endophyte *A. fumigatus* from liverwort *Heteroscyphus tener* (Steph.) Schiffn.s [33]. Fumiquinazoline J (**131**) was also identified from mangrove-derived *A. fumigatus* HQD24 [53]. In addition, **131**, **132**, and fumiquinazoline D (**133**) were obtained from *A. fumigatus* M580 [49]. Compound **131** proved to exert immunosuppression on concanavalin A (ConA)-stimulated T-cell proliferation and LPS-stimulated B-cell proliferation, with IC_50_ values of 29.38 ± 0.21 and 162.58 ± 2.39 μM, respectively. It also displayed cytotoxicity against Huh7 and HT29 cells, with IC_50_ values of 9.7 ± 0.9 and 10.3 ± 0.9 μM, respectively [53]. Compounds **129**, **130,** and **132** showed moderate activity against PC3, with IC_50_ values of 32.2 ± 0.5, 30.1 ± 0.7, and 27.8 ± 0.4 μM, respectively. Compounds **131** and **132** indicated moderate cytotoxic activity against NCI-H460, with IC_50_ values of 26.9 ± 0.6 and 33.4 ± 0.7 μM, respectively [33]. The MIC values of **132** and **133** against *Enterococcus faecalis* were 32 and 32 µg/mL, respectively. The α-glucosidase inhibition ratio of **132** was 13.6% at 100 μg/mL [49].

Detailed chemical investigation of *A. nidulans* MA-143 associated with *Rhizophora stylosa* resulted in the discovery of four new metabolites, aniquinazolines A–D (**134**–**137**). Compounds **134**–**137** showed potent brine shrimp lethality activity, with median lethal dose (LD_50_) values of 1.27, 2.11, 4.95, and 3.42 μΜ, respectively. None of them exhibited cytotoxicity against BEL-7402, MDA-MB-231, HL-60, or K562 cell lines, nor did they display antibacterial activity against *E. coli* or *S. aureus* [54]. Compounds **134**, **135**, **137**, and 14-epi-isochaetominine C (**138**) were obtained from endophyte *A. versicolor* MA-229 from *Lumnitzera racemosa*, and **138** had an inhibiting effect on *Fusarium graminearum*, with an MIC value of 16 μg/mL [55].

A study on *Melia azedarach*-derived *A. fumigatus* LN-4 revealed previously reported metabolites—fumiquinazolines F (**139**), G (**140**), D (**133**), and A (**141**) and tryptoquivaline O (**142**)—as well as a new alkaloid, 3-hydroxyfumiquinazoline A (**143**). Compounds **133, 139, 141**, and **143** possessed antifeedant activities against armyworm larvae, with AFI values of 10%, 30.0%, 45%, and 7.5%, respectively. Furthermore, compounds **139**–**142** exerted weak lethality toward brine shrimps, with LC_50_ values of 55.3, 78.8, 39.7, and 72.8 μg/mL [31].

Quinadoline C (**144**), identified from *Aspergillus* sp. HS02 associated with *Sonneratia hainanensis*, did not show any anti-fungal activity with mango or rubber anthracnose fungus [56].

Two new glucosidated alkaloids, fumigatosides G (**145**) and H (**146**), were separated from the mangrove-derived fungus *A. fumigatus* SAl12 [57].

An extensive investigation of *A. fumigatus* Y0107 derived from the lateral buds of *Crocus sativus* Linn (saffron) resulted in the identification of known alkaloids **130**, **131**, 18-epi-fumiquinazolin C (**147**), fumigatoside F (**148**), 2′-epi-fumiquinazoline D (**149**), and oxoglyantrypine (**150**). Compound **147** had a mild inhibitory effect on *Erwinia* sp. with an MIC value of 100 μg/mL. Other compounds did not show any activity against *A. tumefaciens*, *P. agglomerans*, *R. solanacearum*, or *Erwinia* sp. (MIC > 100 μg/mL) [58].

The marine red algae-derived endophytic fungus *A. creber* EN-602 was studied, yielding three new diketopiperazines: 3-hydroxyprotuboxepin K (**151**), 3,15-dehydroprotuboxepin K (**152**), and versiamide A (**153**), as well as known analogues brevianamide P (**154**), protuboxepin J (**155**), and **156**. Compounds **151**, **154**, and **155** showed ACE inhibition with IC_50_ values of 11.2, 16.0, and 22.4 μM, respectively. Compounds **152** and **153** exhibited different aquatic bacteria inhibition with MIC values in the range of 8 to 64 μg/mL [59].

A chemical study on the mangrove endophyte *Aspergillus* sp. 16-5c led to the discovery of a new alkaloid, aspergiamide F (**157**), along with known metabolites brevianamide M (**158**) and brevianamide N (**159**). The IC_50_ values of compounds **157**–**159** inhibiting α-glucosidase were 267.3, 67.8, and 362.6 μM, respectively. All the compounds were inactive when it came to PTP1B enzyme activity [48].

The endophyte *A. versicolor* from *Nicotiana tabacum* was cultured, producing four new alkaloids, isoaspergillines B–E (**153**, **160**–**162**), as well as the known compounds (1*R*,4*S*)-4-benzyl-1-isopropyl-2,4-dihydro-1H-pyrazino-[2,1-b]quinazoline-3,6-dione (**163**) and protuboxepin K (**164**). Compounds **153**, **160**–**164** exhibited mosaic virus TMV inhibitory activity, with IC_50_ values of 34.8, 37.9, 32.2, 42.4, 39.5, and 35.2 μM, respectively [46].

A study on *Sargassum kjellmanianum*-derived endophyte *A. ochraceus* revealed a new compound, 2-hydroxycircumdatin C (**165**), and two known analogues, circumdatin F (**166**) and circumdatin C (**167**). Compound **165** exhibited obvious 2,2-diphenyl-1-picrylhydrazyl (DPPH) inhibition, with an IC_50_ of 9.9 μM. However, none of them displayed antibacterial activity [41].

The fungus *A. terreus* IFB-E030 collected from *Artemisia annua* was found to generate a new compound, 16α-hydroxy-5N-acetylardeemin (**168**), and two previously reported metabolites, 5N-acetylardeemin (**169**) and 15b-β-hydroxy-5N-acetylardeemin (**170**). Compounds **168**–**170** exhibited AChE inhibitory activity, with IC_50_ values of 58.3, 149.4, and 116.9 µM, respectively, and showed moderate-to-weak cytotoxicity against KB cells, with IC_50_ values of 149.6, 106.7, and 61.4 µM, respectively. Compounds **168** and **170** showed mid inhibitory activity against HSC-T6 cells, with IC_50_ values of 69.2 and 47.3 µM, respectively [26].

Four compounds—**168**–**170** and 5-N-acetyl15b-didehydroardeemin (**171**)—were purified from endophytic fungus *A. fumigatus* SPS-02 harbored by *Artemisia annua* L. Compound **168** reversed MDR in K562/DOX and A549/DDP cell lines with 5.2 ± 0.18-fold, and 8.2 ± 0.23-fold at 5 μM, respectively. Compounds **170** and **171** significantly improved anti-SK-OV-S/DDP cell line activity, with 10.8 ± 0.28-fold, and 8.7 ± 0.21-fold, respectively [60].

**Table 3:** Quinazoline Alkaloids from endophytic fungi of *Aspergillus* genus and their biological activities, metabolite class, fungus, host plant(s), reference.

Fungus	Host Plant(s)	Compounds Isolated	Biological Target	Biological Activity	Reference
*A. flavipes* DZ-3	*Eucommia ulmoides* Oliver	Asperflaloid A (**126**)	α-Glucosidase inhibitory and antioxidant activities	Inactive	[52]
		2-(4-Hydroxybenzyl)quinazolin-4(3H)one (**127**)	α-Glucosidase inhibition	IC_50_ of 750.8 µM	
*Aspergillus* sp.	Moss	Versicomide E (**128**)	Anti-inflammatory activity to suppress NO production in RAW 264.7 cells stimulated by LPS	Inactive	[44]
*Aspergillus* sp. 87/*A. fumigatus*	Mangrove/*Heteroscyphus tener* (Steph.)Schiffn.s	Isochaetominine (**129**)	Antibacterial activities against *Pseudomonas aeruginosa*, *Staphylococcus aureus*, *Acinetobacter baumannii*, and *Escherichia coli*; Cytotoxicity against PC3;	MIC > 100µM; IC_50_ of 32.2 ± 0.5 µM	[35,47,53]
*Aspergillus* sp. TPXq/*A. fumigatus/A. fumigatus* Y0107	*Saussurea medusa/Heteroscyphus tener* (Steph.)Schiffn.s/*Crocus sativus* Linn (saffron)	Chaetominine (**130**)	Cytotoxicity against A549, MCF-7 and PC3	IC_50_ of 0.18 μg/mL, 0.89 μg/mL, 30.1 ± 0.7 µM, respectively	[33,35,53,58]
*A. fumigatus/A. fumigatus* HQD24/*A. fumigatus* Y0107	*Heteroscyphus tener* (Steph.)Schiffn.s/mangrove/*Crocus sativus* Linn (saffron)	Fumiquinazoline J (**131**)	Immunosuppression on ConA-induced T-cell proliferation and LPS-induced B-cell proliferation	IC_50_ of 29.38 ± 0.21 and 162.58 ± 2.39 μM, respectively	[33,53,59]
			Cytotoxicity against Huh7, HT29, NCI-H460 cells	IC_50_ of 9.7 ± 0.9, 10.3 ± 0.9, and 26.9 ± 0.6 μM, respectively	
*A. fumigatus/A. fumigatus* M580	*Heteroscyphus tener* (Steph.)Schiffn.s/cucumber	Fumiquinazoline C (**132**)	Cytotoxicity against PC3, and NCI-H460	IC_50_ of 27.8 ± 0.4, and 33.4 ± 0.7 μM, respectively	[33,49]
			Antimicrobial activity against *Enterococcus faecalis*	MIC of 32 µg/mL	
*A. fumigatus/A. fumigatus* M580/*A. fumigatus* LN-4	*Heteroscyphus tener* (Steph.)Schiffn.s/cucumber/*Melia azedarach*	Fumiquinazoline D (**133**)	Antimicrobial activity against *Enterococcus faecalis*	MIC of 32 µg/mL	[31,49]
			α-Glucosidase inhibition ratio	Inhibition ratio 13.6% at 100 μg/mL	
			Inhibitory activity against armyworm larvae	AFI of 10%	
*A. nidulans* MA-143/*A. versicolor* MA-229	*Rhizophora stylosa/Lumnitzera racemosa*	Aniquinazoline A (**134**)	Brine shrimp lethality activity	LD_50_ of 1.27 μΜ	[54,55]
		Aniquinazoline B (**135**)		LD_50_ of 2.11 μΜ	
*A. nidulans* MA-143	*Rhizophora stylosa*	Aniquinazoline C (**136**)		LD_50_ of 4.95 μΜ	[54]
*A. nidulans* MA-143/*A. versicolor* MA-229	*Rhizophora stylosa/Lumnitzera racemosa*	Aniquinazoline D (**137**)		LD_50_ of 3.42 μΜ	[54,55]
*A. versicolor* MA-229	*Lumnitzera racemosa*	14-Epi-isochaetominine C (**138**)	Inhibiting effect on *Fusarium graminearum*	MIC of 16 μg/mL	[55]
*A. fumigatus* LN-4	*Melia azedarach*	Fumiquinazoline F (**139**)	Inhibitory activity against armyworm larvae	AFI of 30%	[31]
			Lethality toward brine shrimps	LC_50_ of 55.3 μΜ	
		Fumiquinazoline G (**140**)	Lethality toward brine shrimps	LC_50_ of 78.8 μΜ	
		Fumiquinazoline A (**141**)	Inhibitory activity against armyworm larvae	AFI of 40%	
			Lethality toward brine shrimps	LC_50_ of 39.7 μΜ	
		Tryptoquivaline O (**142**)	Lethality toward brine shrimps	LC_50_ of 72.8 μΜ	
		3-Hydroxyfumiquinazoline A (**143**)	Inhibitory activity against armyworm larvae	AFI of 7.5%	
			Lethality toward brine shrimps	LC_50_ of 80.8 μΜ	
*Aspergillus* sp. HS02	*Sonneratia hainanensis*	Quinadoline C (**144**)	Anti-fungi activity with *mango and rubber anthracnose fungus*	Inactive	[56]
*A. fumigatus* SAl12	Mangrove	Fumigatoside G (**145**)	-	-	[57]
		Fumigatoside H (**146**)			
*A. fumigatus* Y0107	*Crocus sativus* Linn (saffron)	18-Epi-fumiquinazolin C (**147**)	Antimicrobial activity against *A. Tumefaciens*, *P. agglomerans*, *R. solanacearum*, *Erwinia* sp.	Inhibition on *Erwinia* sp. with MIC of 100 μg/mL; others MIC > 100 μg/mL	[58]
		Fumigatoside F (**148**)		MIC > 100 μg/mL	
		2′-Epi-fumiquinazoline D (**149**)			
		Oxoglyantrypine (**150**)			
*A. creber* EN-602	Marine red algal	3-Hydroxyprotuboxepin K (**151**)	ACE inhibition	IC_50_ of 11.2 μM	[59]
		3,15-D K (**152)**	Aquatic bacteria inhibition	MIC values from 8 to 64 μg/mL	
		Versiamide A (**153**)		MIC values from 16 to 64 μg/mL	
		Brevianamide P (**154**)	ACE inhibition	IC_50_ of 16.0 μM	
		Protuboxepin J (**155**)		IC_50_ of 22.4 μM	
		156	-	-	
*Aspergillus* sp. 16-5c	Mangrove	Aspergiamide F (**157**)	α-Glucosidase inhibition	IC_50_ of 267.3 μM	[48]
		Brevianamide M (**158**)		IC_50_ of 67.8 μM	
		Brevianamide N (**159**)		IC_50_ of 362.6 μM	
*A. versicolor*	*Nicotiana tabacum*	Isoaspergilline B (**153**)	TMV inhibitory activities	IC_50_ of 34.8 μM	[48]
		Isoaspergilline C (**160**)		IC_50_ of 37.9 μM	
		Isoaspergilline D (**161**)		IC_50_ of 32.2 μM	
		Isoaspergilline E (**162**)		IC_50_ of 42.4 μM	
		(1R,4S)-4-Benzyl-1-isopropyl-2,4-dihydro-1H-pyrazino-[2,1-b]quinazoline-3,6-dione (**163**)		IC_50_ of 39.5 μM	
		Protuboxepin K (**164**)		IC_50_ of 35.2 μM	
*A. ochraceus*	*Sargassum kjellmanianum*	2-Hydroxycircumdatin C (**165**)	DPPH inhibition	IC_50_ of 9.9 μM;	[41]
			Antibacterial activity	Inactive	
		Circumdatin F (**166**)	Antibacterial activity	Inactive	
		Circumdatin C (**167**)			
*A. terreus* IFB-E030/*A. fumigatus* SPS-02	*Artemisia annua*	16α-Hydroxy-5N-acetylardeemin (**168**)	AChE inhibitory activity	IC_50_ of 58.3 µM	[26,60]
			Cytotoxicity against KB cells and HSC-T6 cells	IC_50_ of 149.6 and 69.2 µM	
			Reverse multidrug resistancce (MDR) in K562/DOX and A549/DDP cell lines	Improving 5.2 ± 0.18-fold, and 8.2 ± 0.23-fold at 5 μM	
		5N-acetylardeemin (**169**)	AChE inhibitory activity	IC_50_ of 149.4 µM	
			Cytotoxicity against KB cells	IC_50_ of 106.7 µM	
		15b-β-Hydroxy-5N-acetylardeemin (**170**)	AChE inhibitory activity	IC_50_ of 116.9 µM	
			Cytotoxicity against KB and HSC-T6 cells	IC_50_ of 61.4 and 67.3 µM	
			Improving anti-SK-OV-S/DDP cell line activity	Improving 10.8 ± 0.28-fold	
*A. fumigatus* SPS-02	*Artemisia annua* L.	5-N-acetyl15b-didehydroardeemin (**171**)	Improving anti-SK-OV-S/DDP cell line activity	Improving 8.7 ± 0.21-fold	[60]

“-” not test.

### 3.4. Quinoline Alkaloids

**Figure 5:**
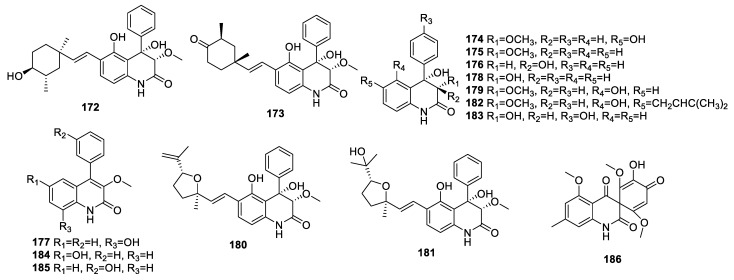
Structures of quinoline alkaloids (**172**−**186**) from endophytic fungi of the *Aspergillus* genus.

A new 4-phenyl-3,4-dihydroquinolone derivative, 22-epi-aflaquinolone B (**172**) (Figure 5, Table 4), together with four related known derivatives, aflaquinolone A (**173**), isoaflaquinolone E (**174**), 6-deoxyaflaquinolone E (**175**), and aflaquinolone G (**176**), were collected from *A. versicolor* MA-229 of *Lumnitzera racemosa*. Compound **172** demonstrated anti-*gaeumannomyces graminis* activity, with an MIC value of 32 μg/mL, and potent *Artemia salina* brine shrimp lethality, with an LD_50_ value of 1.73 μM [55].

Research on *A. creber* EN-602 led to the discovery of **175**, 9-hydroxy-3-methoxyviridicatin (**177**), aflaquinolone F (**178**), and aflaquinolone E (**179**). The MICs of **177** against *Edwardsiella tarda*, *E. coli*, and *Micrococcus luteus* were 64, 32, and 32 μg/mL, respectively [59].

The detailed investigation of *A. nidulans* MA-143 collected from the fresh leaves of *Rhizophora stylosa* revealed new compounds **173**–**175**, aniduquinolones A−C (**180**−**182**), and 14-hydroxyaflaquinolone F (**183**). The LD_50_ values of compounds **173**, **181**, and **182** against brine shrimp (*Artemia salina*) were 5.5, 7.1, and 4.5 μM, respectively. None of them displayed any obvious cytotoxic or antibacterial activity [61].

**Table 4:** Quinoline alkaloids from endophytic fungi of *Aspergillus* genus and their biological activities, metabolite class, fungus, host plant(s), reference.

Fungus	Host Plant(s)	Compounds Isolated	Biological Target	Biological Activity	Reference
*A. versicolor* MA-229	*Lumnitzera racemosa*	22-Epi-aflaquinolone B (**172**)	Anti-*gaeumannomyces graminis* activity	MIC of 32 μg/mL	[55]
			Brine shrimp lethality of *Artemia salina*	LD_50_ of 1.73 μM	
*A. versicolor* MA-229/*A. nidulans* MA-143	*Lumnitzera racemose/Rhizophora stylosa*	Aflaquinolone A (**173**)	Brine shrimp lethality of *Artemia salina*	LD_50_ of 5.5 μM	[55,61]
		Isoaflaquinolone E (**174**)	Antibacterial activity against *Vibrio harveyi*	MIC of 64 μg/mL	
*A. versicolor* MA-229/*A. creber* EN-602/*A. nidulans* MA-143	*Lumnitzera racemose/*marine red algal/*Rhizophora stylosa*	6-Deoxyaflaquinolone E (**175**)	Antibacterial activity against *Vibrio anguillarum*	MIC of 64 μg/mL	[55,59,61]
*A. versicolor* MA-229	*Lumnitzera racemosa*	Aflaquinolone G (**176**)			
*A. creber* EN-602	Marine red algal	9-Hydroxy-3-methoxyviridicatin (**177**)	ACE inhibitory activity	Inactive	[59]
		Aflaquinolone F (**178**)			
		Aflaquinolone E (**179**)			
*A. nidulans* MA-143	*Rhizophora stylosa*	Aniduquinolone A (**180**)	Brine shrimp lethality of *Artemia salina*	Inactive	[61]
		Aniduquinolone B (**181**)		LD_50_ value of 7.1 μM	
		Aniduquinolone C (**182**)		LD_50_ value of 4.5 μM	
		14-Hydroxyaflaquinolone F (**183**)		Inactive	
*Aspergillus* sp	Moss	6-Hydroxy-3-methoxyviridicatin (**184**)	Inhibition on LPS-induced NO production in RAW 264.7 cells	IC_50_ of 22.14 μM	[44]
		3-O-methylviridicatol (**185**)		IC_50_ of 46.02 μM	
*A. fumigatus* CY018	*Cynodon* *dactylon*	Asperfumoid (**186**)	Antimicrobial activity against *Candida albicans*	MIC of 75 μg/mL	[62]

The compounds 6-hydroxy-3-methoxyviridicatin (**184**) and 3-O-methylviridicatol (**185**), identified from *Aspergillus* sp., were found to strongly inhibit NO production induced by LPS in RAW 264.7 cells, with IC_50_ values of 22.14 and 46.02 μM, respectively [44].

The endophyte *A. fumigatus* CY018 obtained from the leaf of *Cynodon dactylon* produced new compound, asperfumoid (**186**), which acted as an antifungal against *C. albicans*, with an MIC of 75 μg/mL [62].

### 3.5. Indole Alkaloids

**Figure 6:**
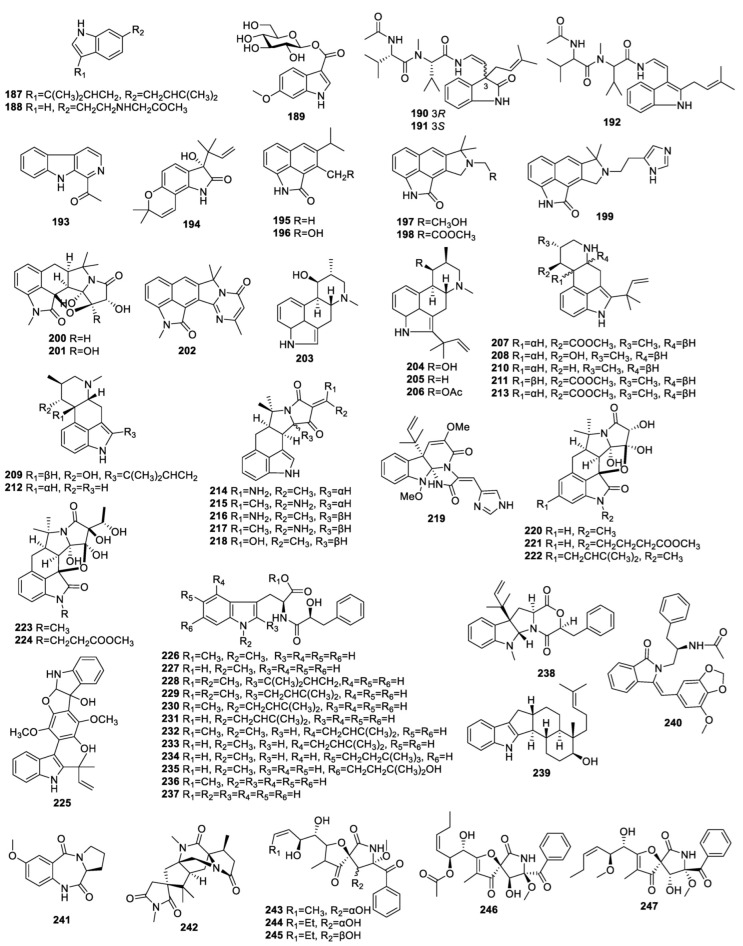
Structures of diketopiperazine alkaloids (**187**−**247**) from endophytic fungi of the *Aspergillus* genus.

Chemical research into *A. amstelodami* generated the compound claudine A (**187**) (Figure 6, Table 5) [38]. A study on *A. fumigatus* from *Erythrophloeum fordii* Oliv. (Leguminosae), resulted in the separation of N-β-lacetyltryptamine (**188**), which did not inhibit NO production [29].

The fungus *A. fumigatus* M580 was investigated, producing a new indole glucoside, named 6-methoxyindole-3-carboxylic acid O-β-D-glucopyranosyl ester (**189**) which did not exhibit inhibition of *C. albicans*, *S. aureus*, *Enterococcus faecalis*, *Salmonella enterica*, or *E. coli* [49].

Two new indolic enamides, terpeptin A (**190**) and B (**191**) and known metabolite **192** were isolated from *Aspergillus* sp. (w-6) growing on *Acanthus ilicifolius*. The IC_50_ values of **190**−**192** against A549 cells were 23.3, 28.0, and 15.0 µM, respectively [40].

1-Acetyl-b-carboline (**193**) was collected from *A. fumigatus* HQD24 associated with *Rhizophora mucronata*. This compound was inactive against HepG2 and conA-induced T-cell proliferation at 10 mg/mL [63].

New alkaloid derivatives, isoaspergilline A (**194**) and aspergillines F–J (**195**–**199**), together with known metabolites aspergilline A (**200**), aspergilline C (**201**), and cyclopiamide E (**202**) were acquired from *A. versicolor* of *Nicotiana tabacum*. Compound **194** exhibited anti-TMV activity with an IC_50_ value of 20.0 μM. Compounds **196** and **199** significantly suppressed TMV, with inhibiting rates of 41.2% and 56.8%, respectively, at 20 μM [46,64].

Fumigaclavine B (**203**), obtained from *A. fumigatus* LN-4 of *Melia azedarach*, showed no toxicity to brine shrimps [31].

Two new metabolites, 9-deacetylfumigaclavine C (**204**) and 9-deacetoxyfumigaclavine C (**205**), along with the known compound fumigaclavine C (**206**), were obtained from *A. fumigatus* of *Cynodon dactylon*. Compound **205** exhibited clear inhibition of K562 cells, with an IC_50_ value of 3.1 µM [65]. Compound **206** was also isolated from *Bauhinia guianensis*-derived *Aspergillus* sp. EJC08 [66], *Heteroscyphus tener* (Steph.) Schiffn-derived *A. fumigatus* [71], mangrove-derived fungus *Aspergillus* sp. 87 [47], and mangrove-derived *A. fumigatus* HQD24 [53]. The bio-assay showed that this compound exhibited cytotoxicity towards PC3, with an IC_50_ value of 26.6 ± 0.7 μM [33], and immunosuppressive activity against T-cell proliferation induced by ConA, with an IC_50_ value of 52.13 ± 0.13 μM [53]. It was devoid of antibacterial activity [47].

A study of the fungus *A. fumigatus* led to the discovery of five new compounds, fumigaclavines D–H (**207**–**211**), and three known isolates, **206**, festuclavine (**212**), and fumigaclavine A (**213**). Compounds **210** and **213** demonstrated clear *Veillonella parvula* inhibition with the same MIC of 16 µg/mL [67].

A detailed study into *A. flavus* GZWMJZ-288 from *Garcinia multiflora* revealed new alkaloids, 19-amino-19-dehydroxy 5-epi-α-cyclopiazonic acid (**214**/**215**) and known analogues 19-amino-19-dehydroxy α-cyclopiazonic acid (**216**/**217**) and α-cyclopiazonic acid (**218**). The IC_50_ values of compounds **214**/**215**, **216**/**217** and **218** for inhibiting α-glucosidase activity were 41.97 ± 0.97, 232.57 ± 11.45, and 243.95 ± 3.36 μM, respectively [68].

The fungus *A. flavipes* DZ-3 of *Eucommia ulmoides* Olive produced the known compound oxaline (**219**), which displayed no antioxidant or α-glucosidase activity [52].

The fungus *A. vesicolor* collected from rhizomes of *Paris polyphylla* var. yunnanensis was studied in depth and generated five new cyclopiazonic acid (CPA) derivatives, aspergillines A−E (**220**−**224**). The IC_50_ values of **220**−**224** for anti-TMV activity were 15.2, 22.8, 41.3, 37.5, and 48.6 μM, respectively. Compound **224** was inactive against PC3, but compounds **220**−**224** had obvious cytotoxicity against NB4, A549, SHSY5Y, PC3, and MCF7 cells, with IC_50_ values ranging from 1.2 to 7.2 μM [69].

Giluterrin (**225**) was produced by the *Axonopus leptostachyus*-derived fungus *A. terreus* P63 and showed inhibitory activity on 786-0, HaCat and PC-3 cells with IC_50_ values of 22.93 ± 8.67, 49.79 ± 10.74 and 48.55 ± 8.06 μM, respectively [70].

The endophyte *A. aculeatus* from *Carica papaya* yielded 10 new alkaloids, aculeatines A–J (**226**–**235**), and known compounds **219**, N-[(2*S*)-2-hydroxy-1-oxo-3- phenylpropyl]-L-tryptophan methyl ester (**236**), N-[(2*S*)-2hydroxy-1-oxo-3-phenylpropyl]-L-tryptophan (**237**), acudioxomorpholine (**238**), and emindole SB (**239**) [36].

A new alkaloid, aspergillinine B (**240**), was generated by *Aspergillus* sp. HAB10R12 of *Garcinia*, which had inactivity against HepG2 and A549 cells [50].

### 3.6. Pyrrolidine Alkaloids

The fungus *A. ochraceus* from *Sargassum kjellmanianum* generated (11a*S*)-2,3-dihydro-7-methoxy-1*H*-pyrrolo[2,1-c][1,4]benzodiazepine-5,11(10*H*,11a*H*)-dione (**241**) (Figure 6, Table 6), which was inactive against *E. coli*, *S. aureus*, and *A. niger* [41]. The fungus *A. aculeatus* associated with *Carica papaya* produced 16-keto-aspergillimide (**242**) [36].

The endophyte *A. fumigatus* was studied and yielded the known metabolites 14-norpseurotin (**243**) and pseurotin A (**244**). Compound **243** promoted the neurite outgrowth of PC12 cells at 10.0 µM [65]. Compound **244** was also produced from *Aspergillus* sp. EJC08 associated with *Bauhinia guianensis* [66], *A. fumigatus* associated with *Erythrophloeum fordii* Oliv [29], *A. fumigatus* associated with *Heteroscyphus tener* (Steph.) Schiffn [33], *A. fumigatus* D associated with *Edgeworthia chrysantha* Lindl [30], and *Aspergillus* sp. 87 [47]. Compound **244** exhibited inhibition of *S. aureus*, *B. subtilis*, *Pseudomonas aeruginosa*, and *E. coli*, with MICs of 15.62, 31.25, 31.25, and 15.62 μg/mL, respectively [66], and inhibited lipopolysaccharide-induced proinflammatory factors in BV2 cells, with an IC_50_ value of 5.20 µM [29].

A chemical study of *A. fumigatus* LN-4 obtained from *Melia azedarach* led to the discovery of **244** and pseurotin A1 (**245**), which demonstrated nontoxicity toward brine shrimps [31].

A new alkaloid, 11-acetyl-pseurotin A2 (**246**), and the known compound 11-O-methylpseurotin A (**247**) were collected from *A. fumigatus* Y0107 of *Crocus sativus* Linn (saffron). These compounds were inactive against *Pantoea agglomerans*, *Agrobacterium tumefaciens*, *Erwinia* sp, and *Ralstonia solanacearum* [58].

### 3.7. Other Alkaloids

**Figure 7:**
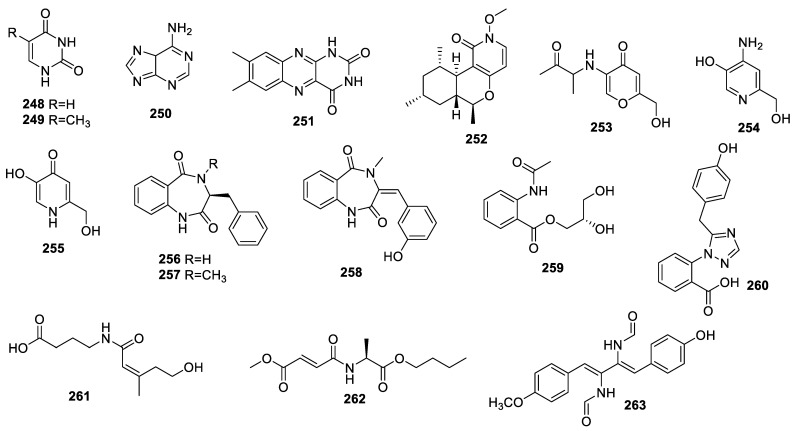
Structures of diketopiperazine alkaloids (**248**−**263**) from endophytic fungi of the *Aspergillus* genus.

A chemical study on *A. amstelodami* revealed compounds **248** (Figure 7, Table 7), thymine (**249**) and adenine (**250**). Compound **248** was also isolated from *A. fumigatus* LN-4 obtained from *Melia azedarach*. Compounds **248** and **250** suppressed melanin production in B16 melanoma cells with IC_50_ values of 144.7 ± 2.35 and 100.4 ± 3.05 µM, respectively [31,38].

The known alkaloid lumichrome (**251**), obtained from *A. fumigatus* collected from *Erythrophloeum fordii* Oliv. (Leguminosae), did not inhibit NO production [29].

The endophyte *Aspergillus* sp. TJ23 collected from leaves of *Hypericum perforatum* (St John’ Wort) yielded a new pyridone alkaloid, asperpyridone A (**252**), which improved glucose uptake in HepG2 cells at 50 μM [71].

New alkaloids 2-Hydroxymethyl-5-(3-oxobutan-2-yl)aminopyran-4(4H)-one (**253**) and 4-amino-2-hydroxymethylpyridin-5-ol (**254**) and the known compound 5-hydroxy-2-hydroxymethylpyridine-4(1H)-one (**255**) were obtained from *A. flavus* GZWMJZ-288 on *Garcinia multiflora*. None of them demonstrated any inhibitory activity against gram-positive *S. aureus* ATCC6538, *S. aureus* ATCC25923, MRSA, gram-negative *Pseudomonas aeruginosa* ATCC10145, or *E. coli* ATCC11775, nor against the pathogenic fungi *C. albicans* ATCC10231 or *C. glabrata* ATCC2001 at 100 μg/mL [68].

A study on *A. creber* EN-602 revealed three previously reported compounds: benzodiazeinedione (**256**), cyclopeptine (**257**), and *trans*-3-(3′-hydroxybenzylidene)-3,4-dihydro-4-methyl-1*H*-1,4-benzodiazepin2,5-dione (**258**). None of them showed any AChE inhibitory activity [59].

A new alkaloid, asperflaloid B (**259**), together with known compounds penipanoid A (**260**), and fuscoatramide (**261**) were obtained from *A. flavipes* DZ-3 of *Eucommia ulmoides* Oliver. These compounds were inactive against antioxidant and α-glucosidase capacities [52].

A new alkaloid, aspergilamide A (**262**), obtained from the fungus *Aspergillus* sp. 87, was devoid of antibacterial activity [47].

N,N’-((1*Z*,3*Z*)-1-(4-hydroxy-phenyl)-4-(4-methoxyphenyl)buta-1,3-diene-2,3-diyl)diformamide (**263**) was isolated from the *A. fumigatus* HQD24, but it did not display inhibition of splenic lymphocyte growth [53].

## 4. Summary and Discussion

**Figure 8:**
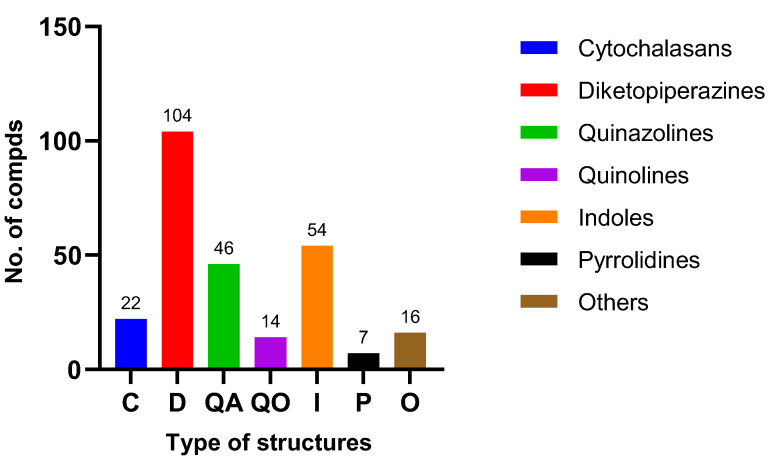
Different classes of Alkaloids from plant endophytic fungi *Aspergillus*.

**Figure 9:**
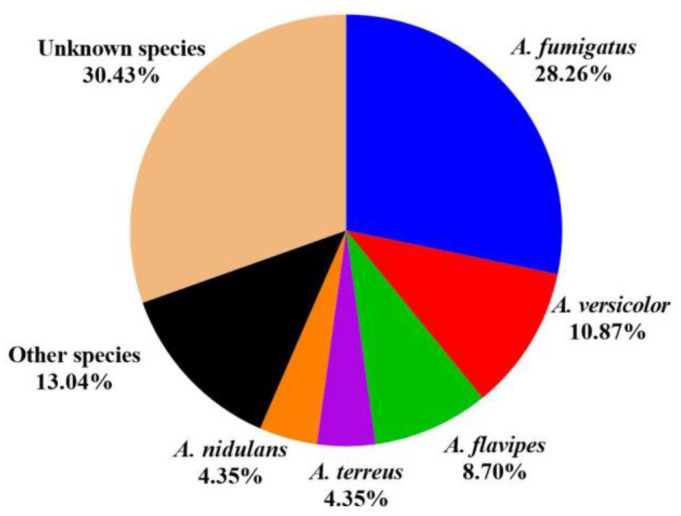
The proportions of *Aspergillus* species reviewed in this paper.

**Figure 10:**
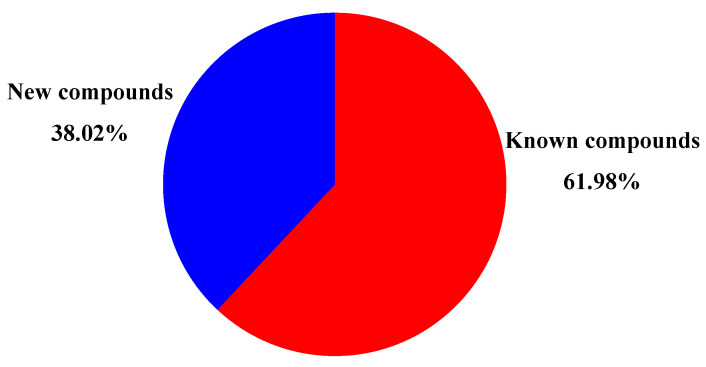
The proportion of new and known alkaloids from plant endophytic fungi *Aspergillus*.

Endophytic fungi are a promising source for novel secondary metabolites. The genus *Aspergillus* is a major reservoir of alkaloids with various structures and diverse bioactivities. In this review, a total of 263 alkaloids derived from endophytic *Aspergillus* (Figure 8), containing 22 cytochalasans, 104 diketopiperazines, 46 quinazolines, 14 quinolines, 54 indoles, 7 pyrrolidines, and 16 others, were acquired from studies on *Aspergillus* genus in the past decades (Figure 8). Among them, diketopiperazine and indole compounds were the main metabolites derived from plant endophytic fungi of the genus *Aspergillus*. All these metabolites were identified from 46 *Aspergillus* strains (Figure 9), of which *A. fumigatus* accounted for 28.26% (13 strains), followed by *A. versicolor* (5, 10.87%), *A. flavipes* (4, 8.70%), *A. terreus* (2, 4.35%), *A. nidulans* (2, 4.35%), other species (6, 13.04%) including *A. aculeatus* (1), *A. amstelodami* (1), *A. cristatus* (1), *A. creber* (1), *A. micronesiensis* (1), and *A. ochraceus* (1), and *Aspergillus* unknown species (14, 30.43%). Detailed analysis revealed that the discovery probability of known alkaloids is high (61.98%) (Figure 10). The microbials inhibited in a special bioenvironment have unique metabolic pathways and potent potential to produce novel bioactive natural products [72]. Therefore, the research of new biological resources is more conducive to the discovery of new biologically active alkaloids. Furthermore, the growing number of *Aspergillus* genome sequences proved that the potential of biosynthetic metabolites is far from having been mined, and bioinformatics analysis revealed that many biosynthetic gene clusters are silent or have low expression under standard laboratory conditions [4,5]. With the development of new research strategies, such as heterologous expression, epigenetic modifiers, and OSMAC, silent and low-expression biosynthetic gene clusters encoding alkaloids in *Aspergillus* might be discovered, and more structurally diverse alkaloids with potent pharmaceutical applications will be found for drug research.

The alkaloids summarized in this literature exhibited antibacterial activity; cytotoxicity; anti-inflammatory activity; and α-glucosidase, ACE, and DPPH inhibitory activities. Many of these metabolites demonstrated potent biological activity. For example, gartryprostatin C (**82**) displayed potent inhibitory activity against the human FLT3-ITD mutant AML cell line MV4-11, with an IC_50_ value of 0.22 μM [39]. Asperpyridone A (**252**) improved glucose uptake and is a potential hypoglycemic agent [71]. However, it is noteworthy that most compounds (114, 43.35%) were inactive in the assays or untested. Further studies for these isolated compounds are necessary to discover their different bioactivity. In addition, some potent active compounds have only been studied in vitro, without further research in vivo and mechanisms of action, which may be limited by the yield of compounds. As we know, some metabolites are generated by endophytic fungi in low quantities under laboratory culture conditions, which make separation difficult and hinder further investigation. Therefore, it requires the interdisciplinary cooperation of chemists, pharmacologists, and biologists to conduct in-depth research on chemical synthesis and modification, as well as genetic regulation to increase the production of active compounds and new analogues, providing chemical research foundation for drug discovery.

## 5. Conclusions

Plant endophytic fungi have provided abundant resources of natural products with unique structural features and diverse biological activities, which play a critical role for drug development. The plant endophytic *Aspergillus* is a dominant community in natural products exploration. In this literature, the bioactivity, structural diversity, and biosources of alkaloids derived from plant endophytic *Aspergillus* species during January 2004 to May 2023 were described. Approximately 263 alkaloids isolated from 46 strains of *Aspergillus* species were reviewed according to their structural features, including cytochalasans, diketopiperazine alkaloids, quinazoline alkaloids, quinoline alkaloids, indole alkaloids, pyrrolidine alkaloids, and others. Among them, 149 alkaloids have significant physiological activities, such as antibacterial activity, cytotoxicity, anti-inflammatory activity, and α-glucosidase, ACE, and DPPH inhibitory activities. Therefore, these active alkaloids have tremendous potential as lead compounds for the exploitation of new drugs. The interdisciplinary research of chemistry, biology, and pharmacology for alkaloids derived from plant endophytic *Aspergillus* sp. has attributed to driving the application of alkaloids in the drug discovery and development.

## Figures and Tables

**Table 1:** Cytochalasans from endophytic fungi of *Aspergillus* genus and their biological activities, metabolite class, fungus, host plant(s), reference.

Fungus	Host Plant(s)	Compounds Isolated	Biological Target	Biological Activity	Reference
*A. micronesiensis*	*Phyllanthus glaucus*	Cytochalasin A (**1**)	Antimicrobial activities against methicillin-resistant *Staphylococcus aureus* (MRSA), *Staphylococcus aureus*, and *Candida albicans*; Cytotoxicities against HL60, human lung cancer A549, Hep3B, MCF-7 and SW480	MIC from 10.6 ± 0.1 to 94.7 ± 1.3 μg/mL; IC_50_ from 3.0 to 19.9 μM	[24]
		Cytochalasin B (**2**)			
		Secochalasin A (**3**)		Inactive	
		Secochalasin B (**4**)			
*Aspergillus* sp.	*Pinellia ternata*	Seco-cytochalasin A (**5**)	Cytotoxic activity against A549	IC_50_ of 55.5 ± 1.87 μM	[25]
		Seco-cytochalasin B (**6**)		IC_50_ of 54.2 ± 1.22 μM	
		Seco-cytochalasin C (**7**)		IC_50_ of 47.2 ± 0.92 μM	
		Seco-cytochalasin D (**8**)		IC_50_ of 40.6 ± 1.30 μM	
		Seco-cytochalasin E (**9**)		IC_50_ of 55.2 ± 1.85 μM	
		Seco-cytochalasin F (**10**)		IC_50_ of 70.2 ± 1.76 μM	
		Cytochalasin Z17 (**11**)		IC_50_ of 58.4 ± 1.78 μM	
*Aspergillus* sp.*/A. terreus* IFB-E030	*Pinellia ternate/Artemisia annua*	Cytochalasin E (**12**)	AChE inhibitory activity; Cytotoxic activity against KB, HSC-T6 and A549 cells	IC_50_ of 146.1 ± 6.5 μM;	[25,26]
				IC_50_ of 113.1 ± 8.3, 47.3 ± 9.9, and 7.8 ± 0.92 μM, respectively	
		Rosellichalasin (**13**)		IC_50_ > 200 μM	
				IC_50_ of 158.3 ± 8.9, >200, and 18.5 ± 1.03 μM, respectively	
*A. terreus* IFB-E030	*Artemisia annua*	5,6-Dehydro-7-hydroxy Cytochalasin E (**14**)	AChE inhibitory activity; Cytotoxic activity against KB and HSC-T6 cells	IC_50_ of 176.0 ± 11.5 μM	[26]
				IC_50_ of 152.9 ± 14.4, >200 μM, respectively	
		Δ^6,12^-Isomer of 5,6-dehydro-7-hydroxy cytochalasin E (**15**)		IC_50_ of 110.9 ± 13.7 μM;	
				IC_50_ of >200 μM and 166.3 ± 13.9 μM, respectively	
*A. flavipes* KIB-536	*Hevea brasiliensis*	Bisaspochalasin A (**16**)	Inhibitory activity against human T cell proliferation	IC_50_ of 15.8 μM	[27]
		Bisaspochalasin B (**17**)		Inactive	
		Bisaspochalasin C (**18**)			
*A. flavipes* KIB-392	*Hevea brasiliensis*	Aspochalasin D (**19**)	_	_	[28]
		Aspochalasin B (**20**)			
		Bisaspochalasin D (**21**)	Cytotoxic activities against HL-60, SMMC-7721, A-549, MCF-7, and SW-480	IC_50_ from 4.45 to 22.99 μM	
		Bisaspochalasin E (**22**)		Inactive	

“_” not test.

**Table 5:** Indole Alkaloids from endophytic fungi *Aspergillus genus* and their biological activities, metabolite class, fungus, host plant(s), reference.

Fungus	Host Plant(s)	Compounds Isolated	Biological Target	Biological Activity	Reference
*A. amstelodami*	White beans	Claudine A (**187**)	-	-	[38]
*A. fumigatus*	*Erythrophloeum fordii* Oliv. (Leguminosae)	N-β-lacetyltryptamine (**188**)	Inhibitory activity of NO production	Inactive	[29]
*A. fumigatus* M580	Cucumber	6-Methoxyindole-3-carboxylic acid O-β-D-glucopyranosyl ester (**189**)	Inhibition on *Candida albicans*, *Staphylococcus aureus*, *Enterococcus faecalis*, *Salmonella enterica*, and *Escherichia coli*	Inactive	[49]
*Aspergillus* sp. (w-6)	*Acanthus ilicifolius*	Terpeptin A (**190**)	Cytotoxic activity against A549 cells	IC_50_ of 23.3 µM	[40]
		Terpeptin B (**191**)		IC_50_ of 28.0 µM	
		192		IC_50_ of 15.0 µM	
*A. fumigatus* HQD24	*Rhizophora mucronata*	1-Acetyl-b-carboline (**193**)	Inhibitory activity against HepG2 and conA-induced T cell proliferation	Inactive at 10 mg/mL	[63]
*A. versicolor*	*Nicotiana tabacum*	Isoaspergilline A (**194**)	Anti-TMV activitiy	IC_50_ of 20.0 μM	[46,64]
		Aspergilline F (**195**)	-	-	
		Aspergilline G (**196**)	Anti-TMV activitiy	Inhibition rate of 41.2% at 20 μM	
		Aspergilline H (**197**)	-	-	
		Aspergilline I (**198**)			
		Aspergilline J (**199**)	Anti-TMV activitiy	Inhibition rate of 56.8% at 20 μM	
		Aspergilline A (**200**)	-	-	
		Aspergilline C (**201**)			
		Cyclopiamide E (**202**)			
*A. fumigatus* LN-4	*Melia azedarach*	Fumigaclavine B (**203**)	Inhibition on brine shrimps	Inactive	[31]
*A. fumigatus*	*Cynodon dactylon*	9-Deacetylfumigaclavine C (**204**)	-	-	[65]
		9-Deacetoxyfumigaclavine C (**205**)			
*A. fumigatus/Aspergillus* sp. EJC08/*A. fumigatus/Aspergillus* sp. 87/*A. fumigatus* HQD24/*A. fumigatus*	*Cynodon dactylon*/*Bauhinia guianen/Heteroscyphus tener* (Steph.) Schiffn/mangrove/mangrove/Cynodon dactylon (Poaceae)	Fumigaclavine C (**206**)	Cytotoxicity for K562 and PC3	IC_50_ of 3.1 and 26.6 ± 0.7 µM, respectively	[33,47,53,65,66,67]
			Immunosuppressive activities against conA-induced T-cell proliferation	IC_50_ of 52.13 ± 0.13 μM	
*A. fumigatus*	*Veillonella parvula*	Fumigaclavine D (**207**)	Antimicrobial activity against *Peptostreptococcus anaerobius*, *Bacteroides diatasonis*, *Veillonella parvula*, *Actinomyces israelii*, *Bacteroides vulgatus* and *Streptococcus anaerobius*	MIC of 64, 64, 32, 64, 128, 128 µg/mL, respectively	[67]
		Fumigaclavine E (**208**)		MIC > 128 µg/mL	
		Fumigaclavine F (**209**)		MIC of 32, 32, 16, 32, 64, 32 µg/mL, respectively	
		Fumigaclavine G (**210**)		MIC > 128 µg/mL, respectively	
		Fumigaclavine H (**211**)		MIC of 32, 32, 16, 32, >128, 32 µg/mL, respectively	
		Festuclavine (**212**)		MIC of 64, 32, 32, 32, 64, 32 µg/mL, respectively	
		Fumigaclavine A (**213**)		MIC of 128, 128, 64, 128, 128, 128 µg/mL, respectively	
*A. flavus* GZWMJZ-288	*Garcinia multiflora*	19-Amino-19-dehydroxy 5-Epi-α-cyclopiazonic acid (**214/215**)	Inhibiting α-glucosidase activity	IC_50_ of 41.97 ± 0.97 μM	[68]
		19-Amino-19-dehydroxy α-Cyclopiazonic acid (**216/217**)		IC_50_ of 232.57 ± 11.45 μM	
		α-Cyclopiazonic acid (**218**)		IC_50_ of 243.95 ± 3.36 μM	
*A. flavipes* DZ-3/*A. aculeatus*	*Eucommia ulmoides* Olive/*Carica papaya*	Oxaline (**219**)	Antioxidant and α-glucosidase inhibitory activities	Inactive	[36]
*A. vesicolor*	*Paris polyphylla* var. yunnanensis	Aspergilline A (**220**)	Anti-TMV activity; Cytotoxic activity of NB4, A549, SHSY5Y, PC3, and MCF7	IC_50_ of 15.2, 3.8, 1.2, 3.4, 2.6, 1.5 μM, respectively	[69]
		Aspergilline B (**221**)		IC_50_, 22.8, 7.2, >10, 5.4, 2.6, 4.5 μM	
		Aspergilline C (**222**)		IC_50_ of 41.3, 1.2, 2.8, 1.5, 2.8, 3.6 μM, respectively	
		Aspergilline D (**223**)		IC_50_ of 37.5, 2.2, 1.5, 3.6, 4.2, 2.9 μM, respectively	
		Aspergilline E (**224**)		IC_50_, 48.6, 4.7, 2.8, 8.2, >10, 6.5 μM	
*A. terreus* P63	*Axonopus leptostachyus*	Giluterrin (**225**)	Inhibitory activity on 786-0, HaCat and PC-3	IC_50_ of 22.93 ± 8.67, 49.79 ± 10.74 and 48.55 ± 8.06 μM, respectively	[70]
*A. aculeatus*	*Carica papaya*	Aculeatine A (**226**)	Cytotoxicity against the L5178Y	Inactive at 10 mg/mL	[36]
		Aculeatine B (**227**)			
		Aculeatine C (**228**)			
		Aculeatine D (**229**)			
		Aculeatine E (**230**)			
		Aculeatine F (**231**)			
		Aculeatine G (**232**)			
		Aculeatine H (**233**)			
		Aculeatine I (**234**)			
		Aculeatine J (**235**)			
		N-[(2S)-2-hydroxy-1-oxo-3-phenylpropyl]-L-tryptophan methyl ester (**236**)			
		N-[(2S)-2hydroxy-1-oxo-3-phenylpropyl]-L-tryptophan (**237**)			
		Acudioxomorpholine (**238**)			
		Emindole SB (**239**)			
*Aspergillus* sp. HAB10R12	*Garcinia scortechinii*	Aspergillinine B (**240**)	Cytotoxicity against HepG2 and A549 cells	Inactive	[50]

“-” not test.

**Table 6:** Pyrrolidine Alkaloids from endophytic fungi of *Aspergillus* and their biological activities, metabolite class, fungus, host plant(s), reference.

Fungus	Host Plant(s)	Compounds Isolated	Biological Target	Biological Activity	Reference
*A. aculeatus*	*Carica papaya*	(11a*S*)-2,3-dihydro-7-methoxy-1*H*-pyrrolo[2,1-c][1,4]benzodiazepine-5,11(10*H*,11a*H*)-dione (**241**)	Antimicrobial activity against *Escherichia coli*, *Staphylococcus aureus*, and *Aspergillus niger*	Inactive	[36]
		16-Keto-aspergillimide (**242**)			[36]
*A. fumigatus*	*Cynodon dactylon*	14-Norpseurotin (**243**)	Activity of promoting neurite outgrowth	Promoting PC12 cells neurite outgrowth at 10.0 µM	[65]
*A. fumigatus/Aspergillus* sp. EJC08/*A. fumigatus/A. fumigatus/A. fumigatus* D/*Aspergillus* sp. 87/*A. fumigatus* LN-4	*Cynodon dactylon/Bauhinia guianensis/Erythrophloeum fordii* Oliv/*Heteroscyphus tener* (Steph.) Schiffn/*Edgeworthia chrysantha* Lindl/mangrove/*Melia azedarach*	Pseurotin A (**244**)	Antimicrobial activity against *Staphylococcus aureus*, *Bacillus subtilis*, *Pseudomonas aeruginosa*, and *Escherichia coli*	Antimicrobial activity with MICs of 15.62, 31.25, 31.25, and 15.62 μg/mL, respectively	[29,30,31,33,47,65,66]
			Anti-inflammatory activity induced by lipopolysaccharide in BV2 cells	Anti-inflammatory activity with IC_50_ of 5.20 µM	
*A. fumigatus* LN-4	*Melia azedarach*	Pseurotin A1 (**245**)	Toxicity toward brine shrimps	Inactive	[31]
*A. fumigatus* Y0107	*Crocus sativus* Linn (saffron)	11-Acetyl-pseurotin A2 (**246**)	Antimicrobial activity against *P. agglomerans*, *A. tumefaciens*, *Erwinia* sp., and *R. solanacearum*	Inactive	[58]
		11-O-methylpseurotin A (**247**)			

**Table 7:** Other Alkaloids from endophytic fungi of *Aspergillus* and their biological activities, metabolite class, fungus, host plant(s), reference.

Fungus	Host Plant(s)	Compounds Isolated	Biological Target	Biological Activity	Reference
*A. amstelodami/A. fumigatus* LN-4	White beans/*Melia azedarach*	**248**	Inhibitory activity on melanin production in B16 melanoma cells	IC_50_ of 144.7 ± 2.35 µM	[31,38]
*A. amstelodami*	White beans	Thymine (**249**)	-	-	
		Adenine (**250**)	Inhibitory activity on melanin production in B16 melanoma cells	IC_50_ of 100.4 ± 3.05 µM	
*A. fumigatus*	*Erythrophloeum fordii* Oliv. (Leguminosae)	Lumichrome (**251**)	Inhibitory activity of NO production	Inactive	[29]
*Aspergillus* sp. TJ23	*Hypericum perforatum* (St. John’s Wort)	Asperpyridone A (**252**)	Activity of glucose uptake in HepG2 cells	Improving glucose uptake in HepG2 cells at 50 μM	[71]
*A. flavus* GZWMJZ-288	*Garcinia multiflora*	2-Hydroxymethyl-5-(3-oxobutan-2-yl)aminopyran-4(4H)-one (**253**)	Inhibitory activity against gram positive *Staphylococcus aureus* ATCC6538, *S. aureus* ATCC25923 and MRSA, gram-negative *Pseudomonas aeruginosa* ATCC10145 and *Escherichia coli* ATCC11775, the pathogenic fungi *Candida albicans* ATCC10231 and *C. glabrata* ATCC2001	Inactive at 100 μg/mL	[68]
		4-Amino-2-hydroxymethylpyridin-5-ol (**254**)			
		5-Hydroxy-2-hydroxymethylpyridine-4(1H)-one (**255**)			
*A. creber* EN-602	*Rhodomela confervoides*	Benzodiazeinedione (**256**)	ACE inhibitory activity	Inactive	[59]
		Cyclopeptine (**257**)			
		*Trans*-3-(3′-hydroxybenzylidene)-3,4-dihydro-4-methyl-1*H*-1,4-benzodiazepin2,5-dione (**258**)			
*A. flavipes* DZ-3	*Eucommia ulmoides* Oliver	Asperflaloid B (**259**)	Antioxidant and α-glucosidase inhibitory activity	Inactive	[52]
		Penipanoid A (**260**)			
		Fuscoatramide (**261**)			
*Aspergillus* sp. 87	Mangrove	Aspergilamide A (**262**)	-	-	[47]
*A. fumigatus* HQD24	Mangrove	N,N’-((1*Z*,3*Z*)-1-(4-hydroxy-phenyl)-4-(4-methoxyphenyl)buta-1,3-diene-2,3-diyl)diformamide (**263**)	Inhibition on splenic lymphocyte growth	Inactive	[53]

“-” not test.

## Data Availability

Not applicable.

## Abbreviations

AChE	Acetylcholinesterase
AFI	Antifeedant indexes
CPA	Cyclopiazonic acid
ConA	Concanavalin A
IC_50_	Half maximal inhibitory concentration
LC_50_	Median lethal concentration
LD_50_	Median lethal dose
LPS	Lipopolysaccharide
MCF7/DOX	Doxorubicin resistant human breast cancer
MDR	Multidrug resistance
MIC	Minimum inhibitory concentration
MRSA	Methicillin-resistant *Staphylococcus aureus*
NO	Nitric oxide
OSMAC	One strain many compounds
PDA	Potato dextrose agar
PTP1B	Protein tyrosine phosphatase 1B
RI	Response index
TMV	Tobacco mosaic virus
Cell lines in the review	
786-0	Renal cell adenocarcinoma
A549	Lung epithelial cell line
A549/DDP	Human lung adenocarcinoma cis-platin resistant
Bel-7402	Papillomavirus endocervical adenocarcinoma
BV2	Microglia
B16	Melanoma cells
HepG2	Hepatocellular carcinoma
Hep3B	Hepatocellular carcinoma
HL-60	Promyelocytic leukemia
HSC-T6	Rat hepatic stellate
HeLa	Human epithelial carcinoma
HT29	Colorectal cancer
Huh7	Hepatoma
HaCat	Human keratinocyte
K562	Myelogenous leukemia
K562/DOX	Leukemia doxorubicin resistant cell
KB	Papilloma epithelial carcinoma
L5178Y	Mouse lymphoblast
MCF-7	Breast ductal carcinoma
MDA-MB-231	Breast epithelial carcinoma
MV4-11	Human acute myeloid leukemi
NCI-H460	Lung giant cell carcinoma
NCM460	Normal colonic epithelial
NB4	Human acute promyelocytic leukemia
PC-3	Prostate adenocarcinoma
PC12	Rat pheochromocytoma
RBL-2H3	Rat basophilic leukemia
RAW 264.7	Murine macrophage
SHSY5Y	Neuroblastoma
SMMC-7721	Hepatocarcinoma
SW-480	Colorectal adenocarcinoma
U-2OS	Human osteosarcoma
